# The landscape of therapeutic vulnerabilities in EGFR inhibitor osimertinib drug tolerant persister cells

**DOI:** 10.1038/s41698-022-00337-w

**Published:** 2022-12-27

**Authors:** Steven W. Criscione, Matthew J. Martin, Derek B. Oien, Aparna Gorthi, Ricardo J. Miragaia, Jingwen Zhang, Huawei Chen, Daniel L. Karl, Kerrin Mendler, Aleksandra Markovets, Sladjana Gagrica, Oona Delpuech, Jonathan R. Dry, Michael Grondine, Maureen M. Hattersley, Jelena Urosevic, Nicolas Floc’h, Lisa Drew, Yi Yao, Paul D. Smith

**Affiliations:** 1 Research and Early Development, Oncology R&D, AstraZeneca, Boston, MA USA; 2 Research and Early Development, Oncology R&D, AstraZeneca, Cambridge, UK; 3 Department of Cell Systems & Anatomy, Greehey Children’s Cancer Research Institute, University of Texas at Health San Antonio, San Antonio, TX USA

**Keywords:** Cancer, Cancer therapeutic resistance, Non-small-cell lung cancer

## Abstract

Third-generation EGFR tyrosine kinase inhibitors (EGFR-TKIs), including osimertinib, an irreversible EGFR-TKI, are important treatments for non-small cell lung cancer with EGFR-TKI sensitizing or EGFR T790M resistance mutations. While patients treated with osimertinib show clinical benefit, disease progression and drug resistance are common. Emergence of de novo acquired resistance from a drug tolerant persister (DTP) cell population is one mechanism proposed to explain progression on osimertinib and other targeted cancer therapies. Here we profiled osimertinib DTPs using RNA-seq and ATAC-seq to characterize the features of these cells and performed drug screens to identify therapeutic vulnerabilities. We identified several vulnerabilities in osimertinib DTPs that were common across models, including sensitivity to MEK, AURKB, BRD4, and TEAD inhibition. We linked several of these vulnerabilities to gene regulatory changes, for example, TEAD vulnerability was consistent with evidence of Hippo pathway turning off in osimertinib DTPs. Last, we used genetic approaches using siRNA knockdown or CRISPR knockout to validate AURKB, BRD4, and TEAD as the direct targets responsible for the vulnerabilities observed in the drug screen.

## Introduction

Single agent targeted cancer therapies frequently induce tumor regression, but rarely result in the elimination of disease due to the emergence of drug resistance^1^. Two non-mutually exclusive mechanisms are frequently proposed to explain drug resistance. The first mechanism is that resistance can emerge from the selection of a pre-existing resistant subclone within a heterogenous tumor. The second mechanism is that drug resistance emerges de novo and is acquired from a therapy insensitive residual disease state^2^. Supporting this second mechanism, long-term drug treatment studies of cancer cell lines or tumor xenografts led to the characterization of drug tolerant persisters (DTPs), a reversible therapy insensitive cell population^3,4^. Exploring DTPs using cancer models can improve our understanding of post-treatment tumors and may identify drug combinations to target therapy insensitive tumors in the clinic.

Approximately 15–20% of non-small cell lung cancer (NSCLC) patient tumors have activating epidermal growth factor receptor (EGFR) mutations^5^, of which exon 19 deletions (E746-A750, ~54%) and L858R mutations (~41%) are predominant^6^. The first-generation EGFR inhibitors erlotinib and gefitinib displayed benefit in patients with these activating mutations, but the duration is limited due to the acquisition of secondary resistance mutations. Osimertinib is a third-generation EGFR inhibitor that was developed to overcome the EGFR T790M gatekeeper resistance mutation, observed in ~60% of patients^6^; and has shown clinical benefit in the first-line and second-line therapy setting^7–9^. Despite this efficacy, development of resistance to osimertinib can still occur, including, notably, via the EGFR C797S resistance mutation at the covalent binding site for osimertinib^10^. However, there is no dominant resistance mechanism analogous to T790M and there is inter- and intra-patient heterogeneity in the range of acquired mutations. Further, ~40–50% of patients that progress on osimertinib do not present with a validated resistance mutation^1^. One potential strategy to delay or abolish resistance is to intervene with combinations that target the therapy insensitive cells and prevent or reduce the chance for de novo acquired resistance to emerge.

Cancer DTPs are characterized as a therapy insensitive cell subpopulation with a slower cell cycle that forms after continuous drug dosing^3^. Beyond EGFR inhibitors, multiple drugs with diverse mechanisms have been shown to induce DTP cells in a wide range of cancer models^11^. One hallmark is that, unlike cells with acquired resistance, DTP cell populations recover following drug withdrawal and respond to drug when rechallenged^3^. Another hallmark of DTPs is they have a distinct chromatin state and are sensitive to drugs targeting epigenetic regulators^3,12^. DTP cell populations are also observed in tumor xenograft studies and DTPs have been proposed as a model to study therapy insensitive cancer cells in patients^4,13^.

Identifying mechanisms of drug tolerance and adaptive resistance is critical to enhance the efficacy of targeted therapies in the clinic. Here we integrated RNA-seq and ATAC-seq to characterize the gene regulatory patterns of osimertinib DTPs. We also conducted systematic drug screens to identify therapeutic vulnerabilities in osimertinib DTPs using either upfront or a sequential dosing strategy. We identified several drugs that displayed sensitivity in osimertinib DTPs including inhibitors targeting MEK, AURKB, BRD4, and TEAD. We subsequently explored pathways associated with these drug screen hits and identified regulatory features that might explain the observed sensitivity. We found osimertinib DTPs downregulate MEK activation gene signatures, whereas MEK compensatory resistance gene signatures were increased. The DTPs displayed increased nuclear YAP localization consistent with Hippo pathway turning off. We also observed features consistent with an epithelial-to-mesenchymal transition and dynamic alterations in chromatin accessibility. Last, we used genetic knockout by CRISPR or knockdown by siRNA to validate AURKB, BRD4, and TEAD as the targets for vulnerabilities identified in the drug screens.

## Results

### Osimertinib DTPs show distinct gene expression changes from acute treatment

**Fig. 1:**
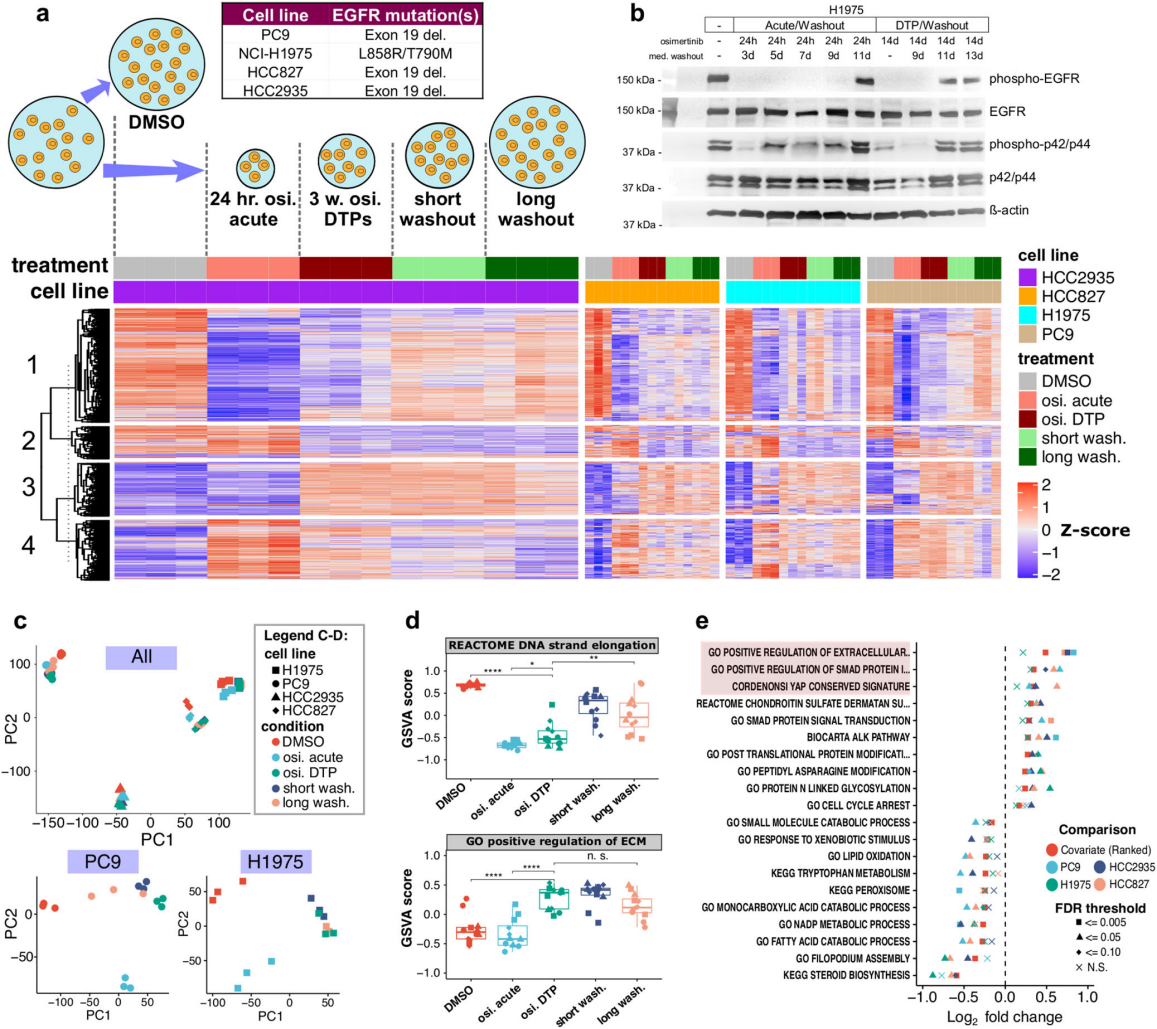
Osimertinib DTPs and acute treatment show distinct gene expression changes. **a** Upper panel: Experimental design of osimertinib DTP RNA-seq time-course. Four EGFR mutant cell lines were treated with DMSO, osimertinib for 24 hours (acute), or osimertinib for 3 weeks (DTPs), followed by short or long washout (see Methods). Lower panel: The top 2000 genes (ranked by FDR) identified to change significantly in 3 of 4 cell line models in any experimental comparison using a moderated F-statistic. Gene expression values were Z-score normalized by cell line and patterns were identified by K-means clustering (K = 4) and subclustered by Euclidean distance. **b** Western blot of phospho-EGFR and phospho-p42/44 in H1975 cells treated with osimertinib acutely for 24 hours or 14 days to form DTPs with or without drug washouts. **c** Upper panel: Principal component analysis (PCA) of normalized log_2_ transcripts per million (TPM) gene expression after removing lowest quantile of least variable genes. Lower panel: Same as **c** upper panel, using only PC9 or H1975 cells. **d** Upper panel: gene set variation analysis (GSVA) scores for Reactome DNA strand elongation pathway (two-sided *t* test, *p* value * < 0.05, ** < 0.01, *** < 0.001). Boxplot is quartiles with range bar as minimum or maximum data values within 1.5 times the interquartile range. Lower panel: Same as **d** upper panel, GSVA scores for GO extracellular matrix pathway. **e** Top-ranked pathway changes in osimertinib DTPs versus acute treatment, selected by lowest FDR of cell line covariate differential GSVA analysis, compared to comparisons done separately in each cell line. The pathways are ordered by cell line covariate log_2_ fold change, color indicates the specific comparison, and shape indicates FDR status.

We profiled osimertinib DTPs in four EGFR mutant, NSCLC cell lines using RNA-seq to identify common gene regulatory changes. Cell lines were treated acutely with osimertinib for 24 hours or treated 21 days to generate DTPs. We also conducted a short-term (24 hours) or long-term (3–10 days chosen by cell line regrowth) drug washout (Fig. 1a upper panel, Supplementary Table 1). Western blot data confirmed that the 21-day treatment led to suppression of phospho-EGFR and phospho-p42/p44. Interestingly, cell regrowth time points chosen for long washouts (3–10 days) occurred before phospho-EGFR and phospho-p42/p44 recovery at 11 days (Fig. 1b). In the RNA-seq, we identified clusters of gene expression changes with similar trends across time (Fig. 1a, lower panel). We found several interesting patterns, such as cluster 3, which contained genes upregulated in osimertinib DTPs, but not in acute treatment, that persisted in drug washout time points (Fig. 1a lower panel, Supplementary Fig. 1A). Cluster 3 genes enriched for epithelial-to-mesenchymal transition (EMT) related pathways including wound healing (Supplementary Fig. 1B). In cluster 1, genes were downregulated in osimertinib acute treatment, gradually recovered in osimertinib DTPs, and continued recovery in drug washout. The pattern in cluster 1 correlated with cell regrowth and enriched for cell cycle and DNA replication genes (Supplementary Fig. 1B). Principal component analysis (PCA) identified that a large source of gene expression variation was from cell line (Fig. 1c upper panel). By performing PCA separately in each cell line, we observed more gene expression variation from drug treatment, with osimertinib DTPs and washout time points grouped separately from DMSO and acute treatment (Fig. 1c lower panel). Clustering and PCA suggested that the DTP selection bottleneck yielded pronounced gene expression changes that differ from acute treatment and persist in drug washout time points.

We further explored differences between acute treatment and osimertinib DTPs (Supplementary Fig. 2). Osimertinib DTPs and acute treatment both displayed decreased cell cycle-related gene expression compared to DMSO (Supplementary Fig. 3A, B). A subset of cell cycle-related pathways and genes returned closer to baseline levels in drug washouts (Supplementary Fig. 3C). The DNA strand elongation pathway, for example, decreased in osimertinib acute treatment, continued suppression in DTPs but to a lesser extent, and recovered gradually in drug washouts (Fig. 1d upper panel, Supplementary Fig. 3D). We also observed distinct pathway changes in osimertinib DTPs versus acute treatment when compared to DMSO, including changes to lysosome and cell metabolism pathways (Supplementary Fig. 3A, B). To further identify alterations specific to DTPs, we directly compared osimertinib DTPs to acute treatment. This comparison displayed more variation between the cell lines (Supplementary Fig. 2C); however, we identified increases to extracellular matrix secretion (ECM) signatures, YAP1 signatures, SMAD signatures, and decreases to cell metabolism pathways (Fig. 1d lower panel, Fig. 1e). Notably, the ECM-related signatures upregulated in DTPs remain high in drug washouts, despite cell-cycle pathways returning closer to baseline levels. This may explain why drug washouts group closer to osimertinib DTPs in PCA, even though they are in the process of regaining proliferative capacity.

### Osimertinib DTPs display chromatin accessibility alterations

**Fig. 2:**
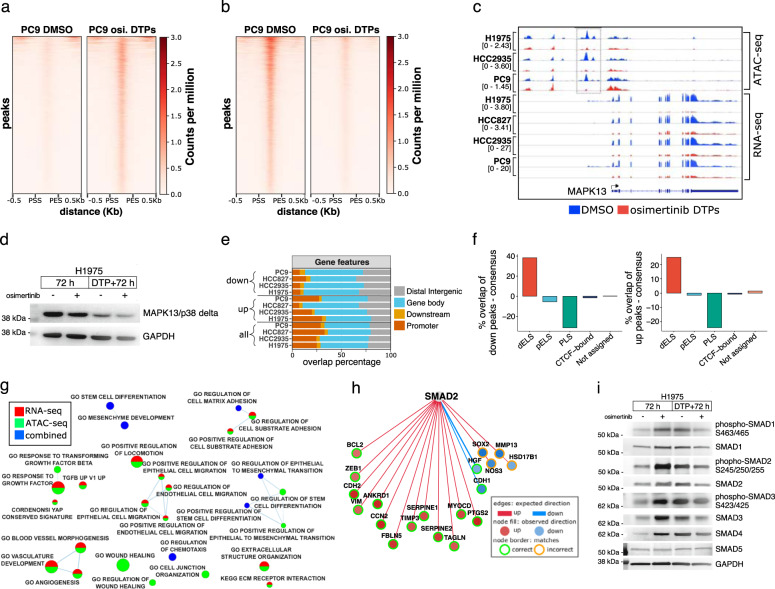
Osimertinib DTPs display altered chromatin accessibility. **a** Significantly increased peaks in PC9 osimertinib DTPs in normalized counts per million (fold change > 2 and FDR < 0.005) in a 500 bp window centered on peak start site (PSS) to peak end site (PES) ± 0.5 kilobases. **b** Same as **a**, significantly decreased peaks in PC9 osimertinib DTPs. **c** Genome browser view of chromatin accessibility decreases in osimertinib DTPs identified upstream of MAPK13 (signal is normalized counts per million). **d** p38δ (MAPK13) Western blot in H1975 cells treated with osimertinib for 72 hours or 3 weeks to form DTPs with or without a 72 hour washout. **e** Proportion of peaks annotated by gene features for all consensus ATAC-seq peaks, peaks increasing significantly, or peaks decreasing significantly. **f** Percent overlap of ENCODE SCREEN regulatory elements for peaks increasing or decreasing chromatin accessibility subtracted by percent overlap in all consensus peaks (dELS distal enhancer like, pELS proximal enhancer like, PLS promoter like). **g** ActivePathways integrated ATAC-seq and RNA-seq cell line meta-analysis identified EMT-related pathways as enriched for significant alterations in osimertinib DTPs versus DMSO (FDR < 0.01). **h** SMAD2 was inferred to increase transcription factor activity by Causal Reasoning (Pollard *p* value = 3.7 × 10^−6^) from gene expression changes in osimertinib DTPs versus acute treatment. The edge color shows expected direction, node fill shows observed direction, and node outline displays whether observed matches expected direction. **i** Western blot of SMAD signaling pathway proteins in H1975 cells treated with osimertinib for 72 hours or 3 weeks with or without a 72 hour washout.

Due to the extensive gene regulatory changes in osimertinib DTPs, we performed ATAC-seq to examine chromatin accessibility. The ATAC-seq data were high quality (Supplementary Table 2); and comparison of peaks across cell lines showed distinct and overlapping chromatin accessibility peaks in the three models profiled (Supplementary Fig. 4A, left panel). Differential peak analysis of osimertinib DTPs versus DMSO identified ~7–15% of peaks either increasing or decreasing in HCC2935, PC9, and H1975 (Supplementary Fig. 4A, right panel). We identified specific regions that gained or lost chromatin accessibility that were significant and reproducible in each cell line (Fig. 2a, b, Supplementary Fig. 4B–G). Regions that decreased accessibility displayed slightly larger effect sizes, suggesting potentially more chromatin silencing in osimertinib DTPs (Fig. 2b). Gained accessibility regions enriched for EMT-related pathways including wound healing, whereas decreased accessibility regions enriched for tyrosine kinase signaling and transcriptional processes (Supplementary Fig. 5A, B). We observed multiple consistent changes across models (Supplementary Fig. 5C), for example, a striking decrease to chromatin accessibility upstream of MAPK13 that was concordant with RNA expression (Fig. 2c, Supplementary Fig. 5D). The gene MAPK13 encodes for mitogen-activated protein kinase p38δ and was confirmed to also decrease at the protein level (Fig. 2d). Globally, decreased accessibility regions enriched at gene body and distal intergenic regions as opposed to promoters, and a similar trend was seen for regions that increased accessibility (Fig. 2e). We also observed higher percent enrichment of distal enhancers, annotated in ENCODE SCREEN, for gained or lost chromatin accessibility regions relative to all peaks (Fig. 2f). These findings suggest that rewiring of enhancer-gene regulation in osimertinib DTPs may drive chromatin-mediated gene expression changes including downregulation of MAPK13.

We next applied ActivePathways^14^, a multiomics method for pathway analysis, to further integrate RNA-seq and ATAC-seq. We also explored Causal Reasoning: a network method to infer upstream regulators from downstream RNA-seq perturbations^15^. When we examined genes upregulated across ATAC-seq and RNA-seq in osimertinib DTPs versus DMSO, we identified multiple interrelated pathways as significantly enriched including ECM and EMT-related pathways, TGF-β pathway, and YAP signatures (Fig. 2g). Interestingly, SMAD2 (Pollard *p* value = 5.11E-05) and SMAD3 (Pollard *p* value = 7.31E-09), were also identified among top-ranked transcription factors inferred to increase activity by Causal Reasoning (Fig. 2h, Supplementary Fig. 5E). The TGF-β/SMAD signaling pathway was previously implicated as a regulator of EMT signaling in lung epithelial cells^16^. In H1975, we found increased total and phosphorylated SMAD1, SMAD2, and SMAD3 by Western blot in osimertinib DTPs (Fig. 2i). We examined EMT marker genes and EMT regulatory transcription factors and observed increases to ZEB1, ZEB2, and SLUG (SNAI2) in H1975 in osimertinib DTPs (Supplementary Fig. 5F). The increase of SLUG in osimertinib DTPs is consistent with previous reports of SLUG increasing in EGFR/MEK inhibited DTPs^13^. We also observed increased levels of expression of mesenchymal marker Fibronectin (Supplementary Fig. 5F). Collectively, these data suggest EMT transition is a feature of osimertinib DTPs and SMAD/EMT-related transcription factors including SLUG (SNAI2) may play a role in this transition.

### Osimertinib DTPs are vulnerable to BRD4, AURKB, and TEAD inhibitors

**Fig. 3:**
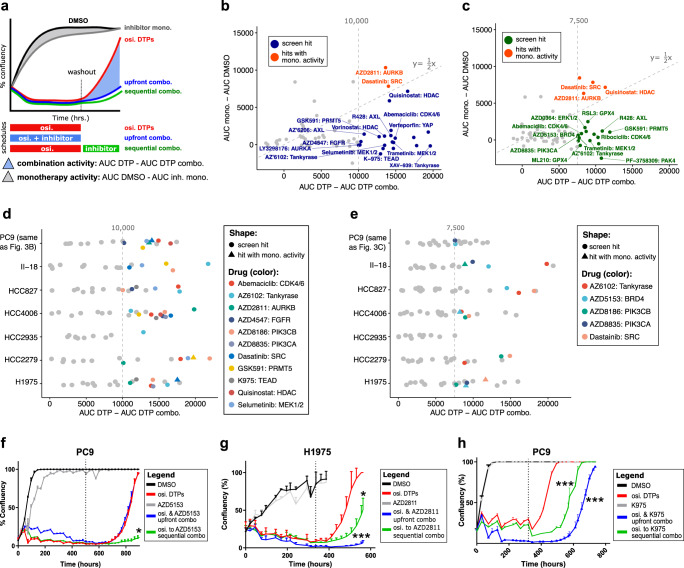
Osimertinib DTP drug combination screens identified vulnerabilities to BRD4, AURKB, and TEAD inhibition. **a** Schematic of osimertinib DTP sequential or upfront drug combination screens. Combination activity was defined as the difference between AUCs for osimertinib DTPs and drug combination DTPs. Hits in the screen were also required to show at least twice the effect of monotherapy activity, defined as the difference between AUC DMSO and AUC monotherapy. **b** PC9 upfront DTP combination screen. Blue highlights screen hits (combination activity >10,000 and >2× monotherapy activity) and red highlights hits with <2× monotherapy activity. **c** Same as **b**, for the PC9 sequential DTP combination screen except >7500 combination activity was used to define hits. **d** Upfront DTP combination screens from PC9 and 6 additional EGFR mutant cell lines. Drugs defined as screen hits (combination activity >10,000 and >2× monotherapy activity) in at least 3 of 7 EGFR mutant cell lines are labeled. For drugs defined as screen hits, we also show if they were a hit with <2× monotherapy activity in another cell line using a triangle shape. **e** Same as **d**, for the sequential DTP combination drug screens. **f** Percent confluency of PC9 cells in upfront or sequential DTP screen with BRD4 inhibitor AZD5153 (300 nM). The dotted line indicates washout in upfront combination or drug crossover in sequential combination and error is s.e.m. The significance is a two-sided *t* test comparing the individual replicate drug combination AUC versus osimertinib monotherapy control (*p* value * < 0.05, ** < 0.005, *** < 0.001). **g** Same as **f**, for AURKB inhibitor AZD2811 (100 nM) in H1975 cells. **h** Same as **f**, for the TEAD inhibitor K-975 (100 nM).

Next, we conducted long-term drug screens in PC9 osimertinib DTPs to identify therapeutic vulnerabilities using compounds with understood pharmacology targeting diverse protein functions in two screen formats (Fig. 3a, Supplementary Data 1–2). In the first screen, we dosed compounds upfront in combination with osimertinib to reduce formation of DTPs. In the second screen, we dosed osimertinib and compounds sequentially to identify dosing regimens that decreased DTP survival. We defined combination activity as the difference in area under the curve (AUC) between osimertinib DTPs and combination regimens. We compared drug combinations to monotherapy activity, defined as the difference between DMSO and drug monotherapy AUC. We filtered for combinations where combination activity in osimertinib DTPs was at least twice the effect of drug monotherapy to define screen hits. In the PC9 upfront screen we observed multiple combinations as hits including AZ6102 and XAV-939 Tankyrase (TNKS) inhibitors, selumetinib and trametinib MEK inhibitors, quisinostat HDAC inhibitor, and K-975 TEAD inhibitors (Fig. 3b). In the PC9 sequential screen we also observed additional drug screen hits including RSL3 and ML210 GPX4 inhibitors, AZD5153 BRD4 inhibitor, and AZ6102 TNKS inhibitor (Fig. 3c). Reassuringly, many of these hits are among previously described vulnerabilities to EGFR inhibitor DTPs including MEK and TEAD inhibitors^13,17^. We next expanded the drug screen, screening six additional EGFR mutant cell lines using inhibitors that showed evidence of some activity in PC9 cells (Supplementary Data 1–2). In the expanded upfront DTP combination screens, we identified common screen hits, in at least three of seven cell lines, including AURKB, CDK4/6, SRC, FGFR, TNKS, PRMT5, HDAC, and TEAD inhibitors (Fig. 3d). In the sequential screen we observed combination activity for TNKS, SRC, PIK3CA/B, and BRD4 inhibitors (Fig. 3e). The sequential screen identified fewer combinations and the strength of activity was less than upfront dosing.

We next closely examined the full drug response patterns. We found that the BRD4 inhibitor AZD5153^18,19^ primarily displayed combination activity when dosed sequentially in PC9, HCC827, and II-18 cell lines, with little or no activity upfront (Fig. 3f, Supplementary Fig. 6A). In contrast, most other hits displayed more activity as an upfront combination including the AURKB inhibitor AZD2811 (Fig. 3g, Supplementary Fig. 6B), the HDAC inhibitor quisinostat (Supplementary Fig. 7A), and the TNKS inhibitor AZ6102 (Supplementary Fig. 7B). Tankyrase inhibitors have been reported to stabilize AMOT, a negative regulator of YAP, and suppress YAP co-factor activity with TEAD^20^. A more direct inhibitor of TEAD, the TEAD inhibitor K975^21^, also displayed combination activity when dosed upfront (Fig. 3h, Supplementary Fig. 7C). We also examined the TEAD inhibitor K-975, BRD4 inhibitor AZD5153, and TNKS inhibitor AZ6102 in a TEAD luciferase reporter assay. We observed potent TEAD reporter activity suppression by K-975, modest suppression by AZD5153 and AZ6102, and no activity for osimertinib and erlotinib negative controls (Supplementary Fig. 7D).

### The BRD4 inhibitor AZD5153 displayed a dose-dependent vulnerability in DTPs

The functional evidence provided by the drug combination screens prompted closer evaluation of gene regulatory changes associated with drug hits. We profiled the sequential treatment of BRD4 inhibitor AZD5153 in osimertinib DTPs using ATAC-seq (Supplementary Fig. 8). In most cell lines AZD5153-treated DTPs displayed additional chromatin accessibility changes, rather than reversing chromatin accessibility alterations observed in DTPs (Supplementary Fig. 8). The only exception was H1975, where AZD5153-treated DTPs showed some reversal of chromatin accessibility decreases in the DTPs, although H1975 was not sensitive to AZD5153 in the drug screen (Supplementary Fig. 8C). Pathway enrichment of increased accessibility regions in AZD5153-treated DTPs indicated changes to EGFR and MEK pathways (Supplementary Fig. 8G), whereas EMT-related pathways including wound healing and apoptosis regulatory pathways decreased (Supplementary Fig. 8H).

We also explored the BRD4 AZD5153 inhibitor dose needed for optimal vulnerability in osimertinib DTPs. In cell lines, we observed a robust vulnerability when DTPs were switched to 100 nM (HCC4006) or 200 nM (PC9) dose of AZD5153 in combination with osimertinib, compared to AZD5153 monotherapy switch or lower dose combination (Supplementary Fig. 9A, B). When we explored the combination in vivo, in an H1975 xenograft model, we observed delayed regrowth when 3 weeks osimertinib treatment was switched to a combination with 2.5 mg/kg AZD5153 (Supplementary Fig. 9C left panel). We rechallenged several tumors from this study and observed they were still sensitive to osimertinib combination with 2.5 mg/kg AZD5153 after drug holiday (Supplementary Fig. 9C right panel). We also examined lower doses of AZD5153 in vivo. In a small study using an EGFR exon 19-deleted patient derived xenograft model (CTG-2531) we observed delayed tumor regrowth using a combination with 0.5 mg/kg AZD5153 (Supplementary Fig. 9D). However, in a larger H1975 xenograft study we observed more variability with a 0.5 mg/kg AZD5153 combination that did not replicate delays in tumor regrowth observed using a 2.5 mg/kg AZD5153 combination (Supplementary Fig. 9E). These observations suggest that the vulnerability of osimertinib DTPs towards the BRD4 inhibitor AZD5153 is dose-dependent.

Next, we tested whether BRD4 genetic knockdown could recapitulate the phenotype observed in osimertinib DTPs. We treated cells with osimertinib to generate DTPs, removed drug, replated surviving cells and transfected with two siRNAs targeting BRD4. We observed a delayed regrowth for DTPs treated with siRNAs targeting BRD4 versus non-targeting control (Supplementary Fig. 10A–C). We also examined apoptotic regulation, a pathway with gene regulatory changes identified by ATAC-seq in AZD5153-treated DTPs (Supplementary Fig. 8H). This led us to identify that AZD5153-treated DTPs upregulated the pro-apoptotic family member BIM (Supplementary Fig. 10D). We tested whether the AZD5153 vulnerability in DTPs was dependent on BIM upregulation using a BIM knockout PC9 model. Using two CRISPR guides targeting BIM, we observed that BIM knockout rescued the decreased cell confluency of AZD5153-treated DTPs (Supplementary Fig. 10E, F). Together these data suggest that the vulnerability of DTPs to AZD5153 is driven by the direct target BRD4, and BIM upregulation plays a role in the vulnerability to BRD4 inhibition.

### The AURKB inhibitor AZD2811 delayed tumor regrowth to osimertinib

**Fig. 4:**
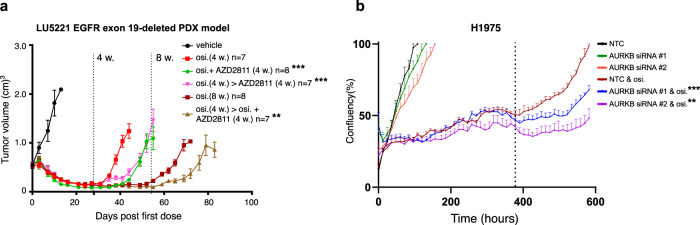
The AZD2811 AURKB inhibitor combination regimens with osimertinib delayed tumor regrowth in vivo. **a** Average tumor volume of a patient derived xenograft LU5221 EGFR exon 19-deleted tumor regrowth model dosed with osimertinib or osimertinib in upfront combination with AZD2811 (dosed IV 25 mg/kg once weekly), or osimertinib monotherapy followed by AZD2811 combination (error is s.e.m. and the legend significance is from a two-sided *t* test versus osimertinib monotherapy endpoint, *p* value * < 0.05, ** < 0.005, *** < 0.001). **b** Percent confluency of an upfront knockdown of AURKB using two siRNAs in combination with osimertinib in H1975 cells (error is s.e.m. and the legend significance is from a two-sided *t* test versus osimertinib non-targeting control endpoint, *p* value * < 0.05, ** < 0.005, *** < 0.001). The dotted line indicates osimertinib washout.

We explored AURKB inhibitor AZD2811 as an upfront hit from the osimertinib DTP drug screen in vivo. We examined an EGFR exon 19-deleted LU5221 PDX model and observed that upfront combinations with osimertinib and AURKB inhibitor AZD2811 (25 mg/kg) or sequential combination delayed tumor regrowth relative to monotherapy (Fig. 4a, Supplementary Fig. 11A). We observed a similar benefit to AZD2811, especially to upfront combination, using a PC9 tumor xenograft study in delaying tumor regrowth (Supplementary Fig. 11B). In an H1975 tumor xenograft study, we observed more variability in tumor regrowth and did not observe delayed tumor regrowth (Supplementary Fig. 11C). We validated the vulnerability was driven by AURKB using knockdown by siRNA of AURKB upfront with osimertinib. We observed that knockdown of AURKB, using two independent siRNAs, led to reduced confluency upon drug removal (Fig. 4b) and verified AURKB knockdown by Western blot (Supplementary Fig. 11D). These findings support AURKB as a vulnerability in osimertinib DTPs, consistent with a recent study that identified AURKB as an EGFR inhibitor resistance target^22^.

### Osimertinib DTPs display altered MEK signaling downstream of EGFR

**Fig. 5:**
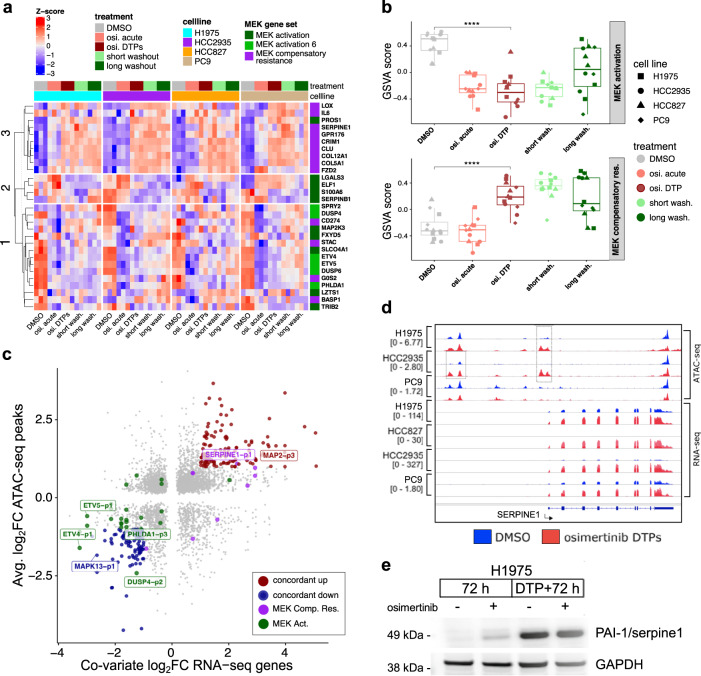
MEK gene signatures display chromatin-mediated gene expression changes in osimertinib DTPs. **a** Gene expression patterns for MEK activation and MEK compensatory resistance genes (MEK activation 6 is a subset of MEK activation^23,24^. Gene expression log_2_TPM values were Z-score normalized by cell line and patterns were identified by K-means clustering (K = 3) and subclustered by Euclidean distance. **b** Upper: GVSA scores of MEK activation genes grouped by treatment (osimertinib DTPs versus DMSO, two-sided *t* test *p* value < 0.001). Boxplot is quartiles with range bar as minimum or maximum value within 1.5 times the interquartile range. Lower: GVSA scores of MEK compensatory resistance genes grouped by treatment (osimertinib DTPs versus DMSO, *p* value < 0.001). **c** Comparison of RNA-seq gene expression fold changes (from cell line covariate analysis) versus consensus peak average fold changes across cell lines that changed significantly in both assays (FDR < 0.005). Concordant up (dark red) genes change at least two-fold up in both assays, concordant down (dark blue) do the opposite. MEK activation genes are dark green and MEK compensatory resistance genes are purple. **d** Genome browser view of SERPINE1, an example MEK compensatory resistance gene, showing coordinated increased chromatin accessibility and increased RNA expression (signal is normalized counts per million). **e** Western blot of PAI-1 (SERPINE1) protein in H1975 cells treated with osimertinib for 72 hours or 3 weeks to form DTPs with or without a 72 hour drug washout.

Next, we examined MEK activation and MEK compensatory resistance signatures^23,24^, because MEK pathway suppression is an expected consequence of EGFR inhibition. The MEK inhibitor selumetinib was also identified in the upfront combination drug screens (Fig. 3d) and MEK inhibitor combinations were previously shown to prevent EGFR inhibitor resistance^13,17,25^. We examined the gene signatures in the RNA-seq and characterized patterns using K-means clustering (Fig. 5a). We found cluster 1, enriched for MEK activation genes that decreased expression in osimertinib acute treatment and DTPs, and trended towards reactivation in drug washouts (Fig. 5a). We also observed cluster 3 enriched for MEK inhibitor compensatory resistance genes that increased in osimertinib DTPs relative to DMSO but did not change in acute treatment (Fig. 5a). Pathway scoring using gene set variation analysis (GSVA) supported a significant decrease in MEK activation signature in osimertinib DTPs, whereas MEK compensatory resistance genes increased significantly (Fig. 5b).

We tested whether chromatin accessibility changes may drive changes to MEK activation and MEK compensatory resistance genes. We compared genes with significant RNA and chromatin accessibility changes across multiple cell lines to identify robust chromatin-mediated gene expression changes in DTPs (Fig. 5c, Supplementary Fig. 12A–C). Several MEK activation genes displayed concordant decreased chromatin accessibility and RNA expression including ETV4, DUSP4, and PHLDA1 (Supplementary Fig. 13A–C). The MEK activation gene DUSP4 also decreased protein levels (Supplementary Fig. 13G). In contrast, the MEK compensatory resistance gene SERPINE1 (PAI-1) displayed concordant increased chromatin accessibility and RNA expression (Fig. 5d) and PAI-1 protein levels also increased (Fig. 5e). Beyond MEK signature genes, this analysis also identified several other concordant changes, including concordant decreases of EPCAM, and conversely increases for MAP2 and IGFBP3 (Supplementary Fig. 13D–F). We confirmed that EPCAM protein decreased, whereas IGFBP3 and MAP2 protein increased (Supplementary Fig. 13G).

Last, we performed ChIP-seq against H3K27ac, a marker of active enhancers and promoters in H1975 cells, as a complementary approach to measure gene regulatory changes. In H3K27ac ChIP-seq data, we identified a very consistent pattern to ATAC-seq with gains and losses of H3K27ac levels in H1975 (Supplementary Fig. 14A, B). Comparisons of H3K27ac versus RNA expression revealed similar patterns to the comparisons with ATAC-seq (Supplementary Fig. 14C). For example, the MEK activation gene DUSP4, like in ATAC-seq, displayed decreased H3K27ac (Supplementary Fig. 14D). Conversely, MEK compensatory resistance gene SERPINE1 displayed a pattern consistent with a super-enhancer, gaining accessibility and increasing H3K27ac levels (Supplementary Fig. 14E). In summary, we identified several chromatin-mediated gene expression changes in osimertinib DTPs that were robust across cell line models, including decreases to MAPK13, EPCAM, DUSP4, PHLDA1, and ETV4 and increases to IGFBP3, MAP2, and SERPINE1. The changes to MEK signatures suggest that upfront combination with a MEK inhibitor may be more effective than sequential dosing; because once the DTPs are established there is an upregulation of MEK compensatory resistance genes. Notably, we did not observe the MEK inhibitor selumetinib as a combination hit in the sequential drug screens.

### Hippo pathway regulatory switch is a characteristic of osimertinib DTPs

**Fig. 6:**
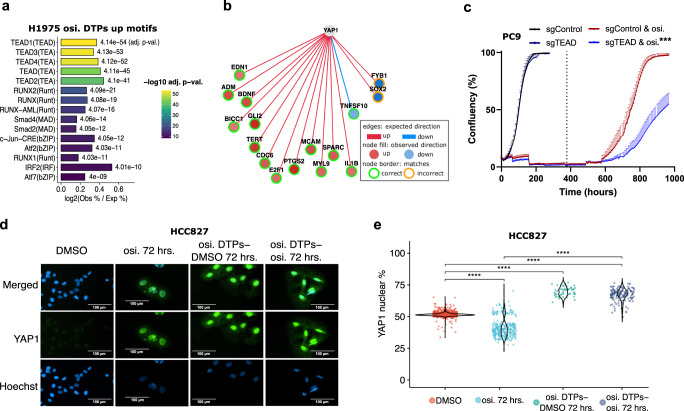
The Hippo pathway turns off in osimertinib DTPs. **a** TEAD transcription factor motifs were enriched for gained accessibility peaks in ATAC-seq (top 15 selected by lowest FDR, label is FDR value) in H1975 osimertinib DTPs**. b** YAP1 was inferred to have increased transcription factor activity in osimertinib DTPs (Pollard *p* value = 0.00056) by Causal Reasoning analysis of genes upregulated in DTPs versus acute treatment (cell line covariate RNA-seq comparison). The edge color shows expected direction, node fill shows observed direction, and the node outline displays whether expected matches the observed direction. **c** PC9 cell confluency for cells treated with osimertinib in combination with upfront knockout of pan-TEAD (CRISPR guide designed against conserved region of TEAD1-4) followed by drug washout (dotted line). Error is s.e.m. and the legend significance is from a two-sided *t*-test versus osimertinib sgControl endpoint (*p* value * < 0.05, ** < 0.005, *** < 0.001). **d** Representative images of YAP nuclear immunofluorescence in HCC827 treated with osimertinib for 72 hours, osimertinib DTPs, or osimertinib DTPs with or without a 72 hour washout. **e** Quantitation of the percentage YAP nuclear immunofluorescence (YAP nuclear/ total YAP) in HCC827 treated with osimertinib for 72 hours, osimertinib for 16 days to form DTPs, or osimertinib DTPs with or without a 72 hour washout (two-sided Wilcoxon signed-rank test, *p* value **** < 0.0001).

In osimertinib DTPs we observed combination activity for TEAD inhibitors and increased YAP gene expression signatures (Fig. 1e, Fig. 3h). To further examine a role for Hippo pathway, we performed transcription factor motif analysis on the ATAC-seq regions that gained or lost chromatin accessibility. In regions that gained accessibility, we identified a robust enrichment of TEAD motifs in all four cell lines (Fig. 6a, Supplementary Fig. 15A, B). We also observed enrichment for GATA-family motifs in PC9 and HCC2935, RUNX-family motifs in H1975, and SMAD-family motifs in H1975 and HCC2935 (Fig. 6a, Supplementary Fig. 15A, B). Decreased accessibility regions displayed more consistency with AP-1 motifs components, such as FOS and JUN, and forkhead motifs, such as FOXA1 and FOXM1, displaying top enrichments across cell lines (Supplementary Fig. 15C–E). Causal Reasoning also identified the TEAD co-factor YAP1 to increase (Pollard *p* value = 0.00056), suggesting increased activity of YAP1 and TEAD in osimertinib DTPs (Fig. 6b).

Causal Reasoning led us to hypothesize that Hippo pathway might turn off in osimertinib DTPs leading to YAP dephosphorylation, translocation to the nucleus, and increased YAP and TEAD activity. Consistent with Hippo pathway turning off, upfront knockout of TEAD using a CRISPR guide targeting a conserved region of TEAD1-4 in DTPs led to decreased confluency after osimertinib withdrawal (Fig. 6c, Supplementary Fig. 15F–H). We also examined YAP protein nuclear localization using immunofluorescence in H1975 and HCC827 osimertinib DTPs. We observed significantly higher percentage of nuclear YAP protein in both HCC827 and H1975 osimertinib DTPs when compared to DMSO (Fig. 6d, e, Supplementary Fig. 15I, J). However, we noted that there were cell line differences in baseline YAP expression. In HCC827 osimertinib DTPs, YAP protein has low baseline expression and total YAP protein is increased, whereas in H1975 total YAP protein only increased modestly from higher baseline levels (Supplementary Fig. 15K). While total YAP levels display cell line differences, percent nuclear YAP protein increased in both cell lines, suggesting more YAP co-factor activity in osimertinib DTPs. As an independent approach, we confirmed in HCC827 that YAP nuclear localization increased, and cytoplasmic localization decreased using subcellular fractionation (Supplementary Fig. 15L). Taken together, these data and the drug screen provide compelling evidence for inactivation of Hippo pathway in osimertinib DTPs resulting in vulnerability to TEAD inhibition.

## Discussion

Drug tolerance in cancer models was characterized by Sharma et al. over a decade ago and the term “drug-tolerant persisters (DTPs)” was used to describe these cells^3^. The DTP cell state was shown to be reversible; DTPs reestablish proliferative capacity after a drug holiday and respond to a drug rechallenge. DTPs are also marked by chromatin-mediated changes and driven by a nongenetic survival mechanism. In the clinical setting, DTPs are hypothesized to be a minimal therapy insensitive disease state that could be a founder cell population for drug resistance^4,26^. A better understanding of osimertinib DTPs could inform rationale selection of drug combinations to drive deeper responses and prevent drug resistance in the clinic.

In this study, we characterized the gene regulatory landscape of osimertinib DTPs using ATAC-seq and RNA-seq and identified therapeutic drug vulnerabilities using drug combination screens. One key component of this study was the use of multiple EGFRm NSCLC models, which enabled identification of robust changes in DTPs. Another distinction was that we could disentangle DTP-specific changes from cell cycle effects by including a 24 hour acute osimertinib treatment in the RNA-seq. In the ATAC-seq, we identified that osimertinib DTPs displayed gains and losses to chromatin accessibility. We observed that decreased chromatin accessibility regions displayed more robust fold changes than gains, consistent with prior studies that described an increased repressive state in DTPs^12^. Guler et al. also described that the increased repressive state of DTPs does not change the total number of unique genes expressed. In our study, this can be explained by increased gene expression observed from SMAD signaling, YAP signatures, and EMT-related pathways while cell cycle and MAPK signaling are turned off.

We identified several genes with concordant chromatin-mediated gene expression changes including genes in MEK signatures. For example, SERPINE1, a MEK compensatory resistance gene was upregulated, whereas the MEK activation genes ETV4, DUSP4, and PHLDA1 were downregulated. The increase in MEK compensatory resistance genes in DTPs may explain why sequential dosing of osimertinib with the MEK inhibitor selumetinib was less effective than upfront drug combinations. Beyond MEK signatures, we identified the gene MAPK13, which encodes p38δ protein, to be concordantly downregulated in osimertinib DTPs. The protein p38δ is an important stress response kinase with context-specific tumor suppressive or tumor promoting roles^27,28^. In line with previous reports that show loss of p38δ might counteract stress-triggered cell death^27,28^, we observed p38δ protein levels decrease in osimertinib DTPs. Overall, we identified multiple robust chromatin-mediated gene expression changes, that could be further validated for potential use as marker genes for in vivo identification of osimertinib DTPs.

Beyond defining concordant gene regulatory changes, we applied integrative network and pathway methods to identify YAP signatures, SMAD signaling, and epithelial-to-mesenchymal transition (EMT) signatures as features upregulated in osimertinib DTPs. Notably, both Hippo pathway and SMAD signaling have previously been described to play a role in EMT phenotypes and cellular plasticity associated with cancer metastasis, so many of these changes may be inter-related^29,30^. Western blot and immunofluorescence data supported the importance of these pathways, with increased levels of phosphorylated SMAD1-3, increased levels of EMT-related transcription factor SLUG (SNAI2) and increased YAP1 nuclear localization found in osimertinib DTPs.

We also performed systematic drug screens for osimertinib DTPs using two assay formats. In the expanded screen we included testing in seven EGFRm NSCLC models. Again, the use of multiple cell lines enabled the identification of inhibitors with robust combination activity. Importantly, the drug screen independently identified several promising combinations with EGFR inhibitors previously reported including AURKB, MEK, TEAD, and TNKS inhibitors^13,17,22^. We validated the AZD2811 AURKB combination benefit in vivo in two of three models tested, with robust delayed tumor regrowth observed in an upfront drug combination. We also found that siRNA knockdown of AURKB reduced cell confluency of osimertinib DTPs indicating combination benefit was likely driven by the direct target of AZD2811.

We also identified sequential drug combination partners with osimertinib, including the BRD4 inhibitor AZD5153. Knockdown of BRD4 also produced a comparable response in DTP cells. Further exploration of combination regimens in vitro found that osimertinib followed by AZD5153 in combination with osimertinib showed the most robust prevention of DTP regrowth. Similar observations were made in vivo using an H1975 xenograft model with delayed tumor regrowth observed using 2.5 mg/kg AZD5153 in combination with osimertinib, but not at lower doses of 0.5 mg/kg AZD5153. The CTG-2531 PDX model, however, responded to a combination of 0.5 mg/kg AZD5153 and osimertinib, which suggests a varied response by model at lower AZD5153 doses. All these monotherapy and combination doses were well tolerated in mice. However, clinical tolerability of AZD5153, up to 30 mg once per day^31^, suggests that the lower doses may be more applicable and that translation of the combination with AZD5153 may be a challenge due to dose-limiting toxicities observed for AZD5153 in the clinic. Together, these efforts identified several promising vulnerabilities, including a dose-dependent vulnerability to BRD4 inhibition, that warrant further study.

Recently, other studies have highlighted the Hippo Pathway as one mechanism underpinning DTP survival, consistent with the activity we observed for the TEAD inhibitor K-975. In melanoma cells, DTP-associated transcriptional changes to BRAF inhibitors were partially mediated by transcription factor activity including TEAD^32^. Kurppa et al. also reported that YAP-mediated transcriptional changes rewired apoptotic pathway to mediate survival of combined EGFR/MEK inhibited DTPs^13^. Kurppa et al. suggest that MEK inhibition combined with EGFR inhibition is needed to observe the changes to the Hippo pathway in DTPs. In our study, EGFR suppression alone seemed to be sufficient to cause a vulnerability to TEAD inhibition in some cell lines. Our data show there may also be cell line variation in the tendency of osimertinib DTPs to rely on Hippo pathway for survival, with differences observed in YAP baseline levels between HCC827 and H1975. Collectively, our study and others highlights TEAD as a vulnerability in osimertinib DTPs and the need for further study of potential crosstalk between Hippo and MAPK signaling.

The DTP state is considered to be an active transition^4^ that is chromatin-mediated and was previously found to be sensitive to HDAC inhibition^3^. Our screens also identified several epigenetic inhibitors as DTP vulnerabilities including the HDAC inhibitor quisinostat and the BRD4 inhibitor AZD5153. This is consistent with an important role for chromatin-mediated changes in regulating the DTP state^33^. We showed using ATAC-seq that AZD5153-treatment induced chromatin accessibility changes in osimertinib DTPs. We also found that BIM upregulation played a role in the decreased cell confluency observed for AZD5153-treated osimertinib DTPs. However, more study is necessary to determine how BRD4-mediated chromatin changes, in certain cell lines such as PC9, cause osimertinib DTPs to upregulate BIM. One interesting observation was that AZD5153 displayed robust combination activity when dosed sequentially. This highlights the need for better time-resolved and single-cell studies to understand the heterogeneity and timing of chromatin-mediated adaptation. Notably, recent single-cell efforts have described metabolic reprogramming in osimertinib DTPs and an increased reliance on fatty acid oxidation^34^. Chromatin modifying enzymes are impacted by the availability of co-factor metabolites, and chromatin and metabolic reprogramming of DTPs may also be linked.

In summary, this study highlights several key gene regulatory features and pathways modulated in osimertinib DTPs and multiple potential therapeutic drug combinations to target DTPs including MEK, TEAD, AURKB, and BRD4 inhibitors that warrant further mechanistic study.

## Methods

### Cell lines

PC9, H1975, HCC827, II-18, HCC4006, HCC2279, and HCC2935 human NSCLC adenocarcinoma cells were obtained from American Type Culture Center and were grown in RPMI 1640, supplemented with 10% FBS, 2 mM L-glutamine, and 1% penicillin-streptomycin. All cells were maintained and propagated as monolayer cultures at 37 °C in a humidified 5% CO_2_ incubator.

### DTP compound screen

The doses of compounds in the screen were selected as the highest dose which did not significantly inhibit the parental cells (>50%) as a monotherapy, or 1 µM, whichever was lowest. To conduct the screen, cells were plated at a density of 20,000–30,000 cells/well in 48-well dishes. The following day (Day 0), cells were treated with compounds at the indicated doses using an HP D300e digital dispenser with the following dosing strategies: DMSO control or (A) test compound monotherapy, (B) osimertinib monotherapy, (C) osimertinib and test compound upfront combination, and (D) osimertinib monotherapy step of a sequential combination. Cell growth was monitored regularly using the IncuCyte live-cell imaging system (Sartorious) using cell confluence as the endpoint. Wells were washed with PBS and replaced with drug-containing medium every 3–4 days to remove dead cells. When the experiment reached the established DTP stage (10–21 days depending on the cell line and experiment), the dosing strategy was altered as follows: (A) switched to drug-free media, (B) switched to drug-free media, (C) upfront combination switched to drug-free media, and (D) sequential combination switched from osimertinib dosing to test compound. When osimertinib monotherapy controls grew to full confluence after drug removal, the experiment was concluded, at which time confluence was plotted and Area Under the Curve (AUC) calculated using PRISM software (Supplementary Data 1). A subset of compounds tested in upfront and sequential screen had experimental triplicates, for these compounds the median replicate AUC was used. Combination activity was defined as the difference between AUC DTPs (B) and AUC DTP combination (C, D). Hits in the screen were also required to show at least twice the effect of monotherapy activity, defined as the difference between AUC DMSO control and AUC monotherapy (A) (Fig. 3a, Supplementary Data 2).

### In vivo tumor studies

Animal studies were conducted in accordance with the AstraZeneca Global Bioethics policy or Institutional Animal Care and Use Committee guidelines and reported following the ARRIVE (Animal Research: Reporting In Vivo experiments) guidelines^35^. For all studies, mice were older than 5 weeks at time of the study.

The 2.5 mg/kg AZD5153 BRD4 inhibitor study was performed as follows: 4 × 10^6^ H1975 cells in 50% Matrigel (Corning) were implanted subcutaneously (SC) into female NCr mice (Charles River Laboratory, US). Tumors were allowed to grow to an average volume of 125 mm^3^ before randomization into treatment groups. Tumor volume measurements were collected over time as described previously^36,37^. Briefly we collected tumor volume as follows: tumor volume was monitored twice weekly by bilateral calliper measurements and volume was calculated using ellipsoid volume formula (π/6 × width^2^ × length). AZD5153 was formulated in 0.5% HPMC/0.1% Tween80 and administered by oral gavage once daily (QD) at 10 mL/kg final dose volume for monotherapy and 5 mL/kg final dose volume for combination treatment. Osimertinib was formulated in 0.5% HPMC and administered by oral gavage once daily (QD) at 10 mL/kg final dose volume for monotherapy and 5 mL/kg final dose volume for combination treatment.

The LU5221 study was performed as follows: Female NSG (NOD.Cg-Prkdcscid Il2rgtm1Wjl/SzJ) mice were purchased from Jackson Laboratories (Bar Harbor ME). LU5221 EGFR Exon 19-deleted non-small cell lung cancer PDX model was procured from Crown Bioscience. The LU5221 PDX model is passaged by dissociation utilizing Miltenyi Human Tumor Dissociation Kits (Miltenyi no. 130-095-929) and a Miltenyi gentleMACS Octo Tissue Dissociator (Miltenyi no. 130-096-427). Xenografts were established using 5 × 10^5^ LU5221 tumor cells suspended in a mixture of PBS and Matrigel (50/50) and implanted subcutaneously (SC) into the right flank of female NSG mice. Mice were randomized based on tumor volumes and enrolled when mean tumor size reached ~500 mm^3^. Osimertinib was formulated in 0.5% HPMC, adjusting for salt content and administered by oral gavage once daily (QD) at 10 mL/kg final dose volume. AZD2811 was formulated in saline and administered intravenously once weekly (QW) at 5 mL/kg final dose volume. Tumor growth was monitored twice weekly as was described in the previous study.

All other studies were performed as follows: PC9 and H1975 xenografts were established by subcutaneous implantation of 5 × 10^6^ cells per animal, in 100 µl of cell suspension including 50% Matrigel, into the dorsal left flank of female SCID or nude mice, respectively. In the CTG-2531 (Champions Oncology) PDX models, tumor fragments from donor mice inoculated with primary human lung cancer tissues were harvested and inoculated subcutaneously into the left of female nude mice. For PC9, H1975, and CTG-2531 studies tumor growth was monitored twice weekly as was described in the study above. Mice were randomized based on tumor volumes and enrolled when mean tumor size reached ~200 mm^3^. For AZD5153 and osimertinib treatments, mice were dosed as described above, except a final dose of 0.5 mg/kg AZD5153 was used instead. For AZD2811 studies, mice were dosed daily by oral gavage for the duration of the treatment period with vehicle, 25 mg/kg osimertinib and combination regimens used a weekly intravenous gavage for the duration of the treatment period with 25 mg/kg weekly AZD2811 nanoparticle.

### RNA-seq

The NSCLC EGFR mutant cell lines (PC9, H1975, HCC827, and HCC2935) were treated with 500 nM osimertinib for 21 days (osimertinib DTPs). Cells were either harvested immediately or washed twice with PBS and then replaced with drug-free media for a further 24 hours (short washout), or for a longer time point (long washout) until exponential cell proliferation resumed (long washout PC9: 7 days; H1975: 3 days; HCC827: 4 days; HCC2935: 10 days) and then harvested. In parallel, parental cell lines were grown in drug-free media for 21 days then treated with 500 nM osimertinib for 24 hours (osimertinib acute) or vehicle DMSO control for 24 hours (DMSO control). Cells were lysed in RLT buffer (Qiagen), and RNA extracted using the Qiacube HT according to manufacturer’s instructions, and RNA concentration quantified using the Qubit fluorometer. Illumina mRNA TruSeq library was used and sequenced on an Illumina HiSeq 4000 with paired-end 150 bp reads by the Cancer Research UK Genomics Core Facility.

### ATAC-seq

The NSCLC EGFR mutant cell lines (PC9, H1975, HCC827, and HCC2935) were treated with 500 nM of osimertinib for 24 days (osimertinib DTPs) or treated with 500 nM of osimertinib for 21 days followed by 150 nM of AZD5153 for 3 days (osimertinib DTP AZD5153 sequential combination). In parallel, parental cell lines were grown in drug-free media for 21 days then treated with vehicle DMSO control for 3 days (DMSO control). ATAC-seq was performed as described previously^38^. Briefly we analyzed ATAC-seq as follows: 100,000 cells were cryopreserved in media containing 10% DMSO and FBS. Cryopreserved cells were sent to ActiveMotif to perform ATAC-seq. Cell pellets were resuspended, spun to pellet, and subsequently tagmented using the Nextera Library Prep Kit (Illumina). The MinElute PCR purification kit (Qiagen) was used to purify the tagmented DNA. The tagmented DNA was amplified using 10 cycles of PCR. Agencourt AMPure SPRI beads (Beckman Coulter) were used to purify the amplified DNA. The DNA yield was measured by KAPA Library Quantification Kit (KAPA Biosystems) and then the ATAC-seq libraries were sequenced with 42 bp paired-end reads on an Illumina NextSeq 500.

### ChIP-seq

H1975 cells were treated with 500 nM of osimertinib with same treatment regimen and controls as was done for ATAC-seq. Cells (10 million) were fixed with 1% formaldehyde for 15 min and quenched with 0.125 M glycine. Cells pellets were sent to Active Motif to perform the ChIP-seq assay using their standard protocol. Briefly, chromatin was isolated by the addition of lysis buffer, followed by disruption with a Dounce homogenizer. Lysates were sonicated and the DNA sheared to an average length of 300–500 bp. Genomic DNA (Input) was prepared by treating aliquots of chromatin with RNase, proteinase K and heat for de-crosslinking, followed by ethanol precipitation. Pellets were resuspended and the resulting DNA was quantified on a NanoDrop spectrophotometer. Extrapolation to the original chromatin volume allowed quantitation of the total chromatin yield.

An aliquot of chromatin (30 µg) was precleared with protein A agarose beads (Invitrogen). Genomic DNA regions of interest were isolated using 4 µl of antibody against H3K27ac (Active Motif cat# 39133, lot# 16119013). Complexes were washed, eluted from the beads with SDS buffer, and subjected to RNase and proteinase K treatment. Crosslinks were reversed by incubation overnight at 65 °C, and ChIP DNA was purified by phenol-chloroform extraction and ethanol precipitation. Quantitative PCR (qPCR) reactions were carried out in triplicate on specific genomic regions using SYBR Green Supermix (Bio-Rad). The resulting signals were normalized for primer efficiency by carrying out qPCR for each primer pair using Input DNA. lllumina sequencing libraries were prepared from the ChIP and input DNA by the standard consecutive enzymatic steps of end-polishing, dA-addition, and adaptor ligation. After a final PCR amplification step, the resulting DNA libraries were quantified and sequenced with 75 bp single-end reads on an Illumina NextSeq 500.

### Western blots

Cell lines were treated with osimertinib for 72 hours or 2–3 weeks to form DTPs with and without a 72 hour drug removal washout. Cells were scraped and lysed with 1% SDS buffer or by NE-PER Nuclear and Cytoplasmic Extraction Reagents (ThermoFisher) when subcellular fractions were done. Protein lysates were normalized after BCA protein quantification (ThermoFisher) and combined with NuPage containing Sample Reducing Agent (ThermoFisher) sample buffer. Protein lysates were separated in 4–20% TGX gels (Bio-Rad), transferred to PVDF membrane (Invitrogen), blocked, and probed overnight with primary antibodies (Supplementary Table 3). Bands were visualized with secondary antibodies (Li-Cor, Lincoln, NE) and imaged using an Odyssey Fc system (Li-Cor), or by HRP secondary antibodies (Cell Signaling) imaged using an Amersham 600 Imager (GE Healthcare) or with Amersham Hyperfilm. All blot sets were derived from the same experiment and processed in parallel. The uncropped Western blots are contained in Supplementary Figs. 16–19. Western Blot antibody vendor, item number, and dilutions are available in Supplementary Table 3.

### Immunofluorescence

Parental cells were seeded next to DTP cells after 2–3 weeks generation in a CellCarrierUltra 96-well plate (PerkinElmer). DTP cells and parental cells were then either dosed for 72 hours with osimertinib or DMSO vehicle. Plates were fixed with paraformaldehyde and blocked with 1% BSA/0.3% TritonX-100 in PBS. Primary antibodies for YAP Alexa Fluor 488 (Abcam) were added at 1:100 dilution for cold overnight incubation. Nuclei were visualized by Hoechst 33342 stain (Abcam). Protein detection was imaged by an Operetta CLS High-Content Analysis System (PerkinElmer) and nuclear versus cytoplasm fractions were quantified using the Harmony 4.9 software. Nuclear YAP percentage was calculated as the percent nuclear YAP fraction versus total nuclear and cytoplasmic YAP signal for individual cells.

### RNA-seq data analysis

RNA-seq was analyzed as previously described^39^. Briefly we analyzed RNA-seq as follows: we used the toolkit bcbio (https://github.com/bcbio/bcbio-nextgen) to implement an RNA-seq analysis pipeline using human hg38 reference genome and hg38 Ensembl transcripts (Ensembl version 79). We aligned RNA-seq data to human hg38 reference genome using HISAT2 (ver. 2.1.0)^40^. Standard data quality metrics were examined with FastQC (https://github.com/s-andrews/FastQC) and multiQC^41^. All experimental samples passed standard data quality metrics (Supplementary Table 1). Ensembl transcripts were quantified from RNA-seq reads using Salmon (ver. 0.8.2)^42^. The R package tximport was used to create a gene by sample matrix of salmon gene counts (using option countsFromAbundance = “lengthScaledTPM”) and a log_2_ transcripts per million, log_2_(TPM + 0.01), abundance matrix^43^. The tximport step aggregated protein coding gene RNA expression with detectable expression in at least two samples.

The Voom and Limma R package were used to conduct differential gene expression analysis on the gene counts matrix^44^. Differential pathway analysis on RNA-seq gene expression was performed using gene set variation analysis (GSVA) and Limma R package using a curated subset of the Molecular Signatures Database (MSigDB: C2, C3, C5, and C6)^45,46^. For hypergeometric pathway enrichment clusterProfiler was used instead^47^. For both differential gene and pathway analysis we compared drug treatments using experimental contrasts within each cell line or across cell lines using a covariate (with model equation ~0 + treatment + cell line). The analysis determines log2 fold changes and FDR adjusted *p* values for genes or pathways within a cell line, or in the case of covariate analysis across cell lines. For the cell line comparisons, we also looked for genes that changed significantly in any comparison using a moderated F-statistic as described in the Limma vignette.

### ATAC-seq data analysis

ATAC-seq analysis was performed using the toolkit bcbio (https://github.com/bcbio/bcbio-nextgen) with the hg38 reference genome. Briefly, reads were aligned to hg38 using bwa mem (version 0.7.17). Mitochondrial reads were removed, and alignments were sorted (samtools v1.9), deduplicated (biobambam v2.0.87 bamsormadup), and tn5-shifted (deeptools v3.4 alignmentSieve). ATAC-seq data quality was addressed with FastQC (https://github.com/s-andrews/FastQC), multiQC, and ataqv^41,48^ (Supplementary Table 2). Fragments smaller than 100 bp were extracted to generate a nucleosome free (NF) alignment bam file used for consensus peak calling. NarrowPeaks were called using MACS2 (v2.2.6) for both NF aligned reads and separately for all aligned reads^49^. Consensus peaks were determined as described in Corces et al.^50^. Briefly, for all NF NarrowPeaks, a 500 bp window was selected around each peak summit. A set of non-overlapping consensus peaks were determined using the maximal scoring peaks as calculated by MACS2 (BEDOPS v2.4^51^). Consensus peak counts were then determined using featureCounts (v2.0.0) counting mapped reads under consensus peak regions^52^. Consensus peak counts were filtered by requiring a maximum log_2_ counter per million ≥ 2 in any experimental group and peaks called in triplicate in at least one experimental group (removing low depth and non-reproducible peaks). Lastly, we annotated peaks with nearest gene using ChIPseeker and focused downstream analysis on peaks within 100 kilobase pair of a gene transcription start site^53^. Bigwig signal files of normalized counts per million were also generated using deeptools bamCoverage (v3.4)^54^.

### ChIP-seq data analysis

ChIP-seq analysis was performed as described for ATAC-seq with the following changes, alignments were not Tn5-shifted, and all alignments were used for MACS2 NarrowPeaks peak calling with IgG sample as a control.

### Downstream and integrative analysis of ChIP/ATAC-seq

Differential peak analysis of ChIP-seq and ATAC-seq was performed using the Voom and Limma R package^44^. Cross cell line meta-analysis of H1975, PC9, and HCC2935 differential ATAC-seq peaks was done by intersecting the differential results for individual cell lines using Genomic Ranges^55^. Intersected meta-analysis of ATAC-seq peaks were summarized, for peaks changing consistently across the three cell lines, by their average fold change and combined adjusted *p* value using Fisher’s method. We compared ChIP-seq and ATAC-seq to RNA-seq by comparing the fold changes of significant differential peaks, to the fold changes of significantly changing gene expression, for the nearest gene. Pathway analysis on ATAC-seq was done using hypergeometric test using ClusterProfiler^47^. Comparison of differential or consensus peaks to ENCODE SCREEN repository of regulatory elements was done by performing overlaps using Genomic Ranges^56^.

### Causal reasoning analysis

Causal Reasoning analysis was implemented using a commercially available R toolkit called Computational Biology Methods for Drug Discovery (CBDD, Clarivate Analytics, v16.1.0)^57^. MetaBase, a manually curated database (also from Clarivate Analytics) was used as the knowledgebase for causal inference. The top 500 differentially expressed genes, ranked by lowest FDR value in the osimertinib DTPs versus acute comparison were used as input. Transcriptional regulators immediately upstream of the observed gene expression changes (one level up from downstream gene) were prioritized from causal reasoning analysis.

### ActivePathway analysis

The list of differentially expressed genes (RNA-seq) or differentially accessible peaks (ATAC-seq) in the osimertinib DTP vs. DMSO comparison were filtered to an effect size of at least two-fold change and adjusted *p* value of less than 0.005. The resulting lists were merged and used as input to ActivePathways versus a curated list of pathway signatures (MSigDB: C2, C3, C5, and C6)^14^. Results were visualized using Cytoscape^58^.

### TEAD reporter assay

Commercially available MCF7 cells containing a luciferase TEAD reporter were acquired from BPS Bioscience and used per the vendor’s instructions. Briefly, cells were seeded in white 96-well plates and incubated overnight. Cells were treated with drugs as indicated for 72 hours. Cells were lysed and luciferase was detected using Bright-Glo (Promega) and detected with a Neo Synergy plate reader (BioTek).

### Knockout/knockdown validation

CRISPR Cas9 (Life Technologies) and TEAD gRNA (5’-TCAGACGAGGGCAAGATGTA-3’ custom sequence covering TEAD1-4) or BCL2L11 gRNA (#1 5’-TTCTGATGCAGCTTCCATG-3’, #2 5’-GCAGGTTCAGCCTGCC-3’), BRD4 siRNAs (Horizon Discovery LQ-004937-00-0020, siRNA#1: J-004937-06 and siRNA#2: J004937-08, targeting BRD4 Entrez ID 23476, sequences not disclosed), or AURKB siRNA (s17611 4392421 targeting CTACAACTATTTTTATGACCG and s17612 4390826 targeting GTGGACCTAA AGTTCCCCGCT, Ambion) were added to cells prior to electroporation. For BRD4, DTP cells were generated by 16-day 500 nM osimertinib treatment prior to adding reagents and electroporation. Electroporation was performed in a 4D Nucleofector (Lonza) with the EN-150 program and SG buffer. Cells were diluted in recovery medium and seeded into 96-well plates for IncuCyte experiments or 6-well plates for efficiency detection.

### Reporting summary

Further information on research design is available in the Nature Research Reporting Summary linked to this article.

## Supplementary information

Supplementary Material

Supplementary Data 1

Supplementary Data 2

REPORTING SUMMARY

## Data Availability

The RNA-seq, ATAC-seq, and ChIP-seq data used in this study are available through the NCBI GEO database under accession code GSE193259. The remaining data used in this study, including drug screens results, are available in the supporting Supplementary Data.

## Code availability

No custom code was used in this study. RNA-seq was analyzed using bcbio (v1.2.3), HISAT2 (v2.1.0), Salmon (v0.8.2), and Limma, Voom, ActivePathways, and ClusterProfiler R packages. ChIP-seq and ATAC-seq were analyzed using bcbio (v1.2.3), bwa mem (v0.7.17), samtools (v1.9), biobambam (v2.0.87), deeptools (v3.4), MACS2 (v2.2.6), BEDOPS (v2.4), featureCounts (v2.0.0), ChIPseeker (v1.29.1) and Limma, Voom, ActivePathways, and ClusterProfiler R packages. All statistical analyses were performed in R version 4.1.1.
