# Supplementary material for: The landscape of therapeutic vulnerabilities in EGFR inhibitor osimertinib drug tolerant persister cells
Source: NPJ Precis Oncol. 2022 Dec 27;6:95. doi: 10.1038/s41698-022-00337-w (PMC9794691; doi:10.1038/s41698-022-00337-w)
Supplement: Supplementary file 1 — Supplementary Material [file 41698_2022_337_MOESM1_ESM.pdf]

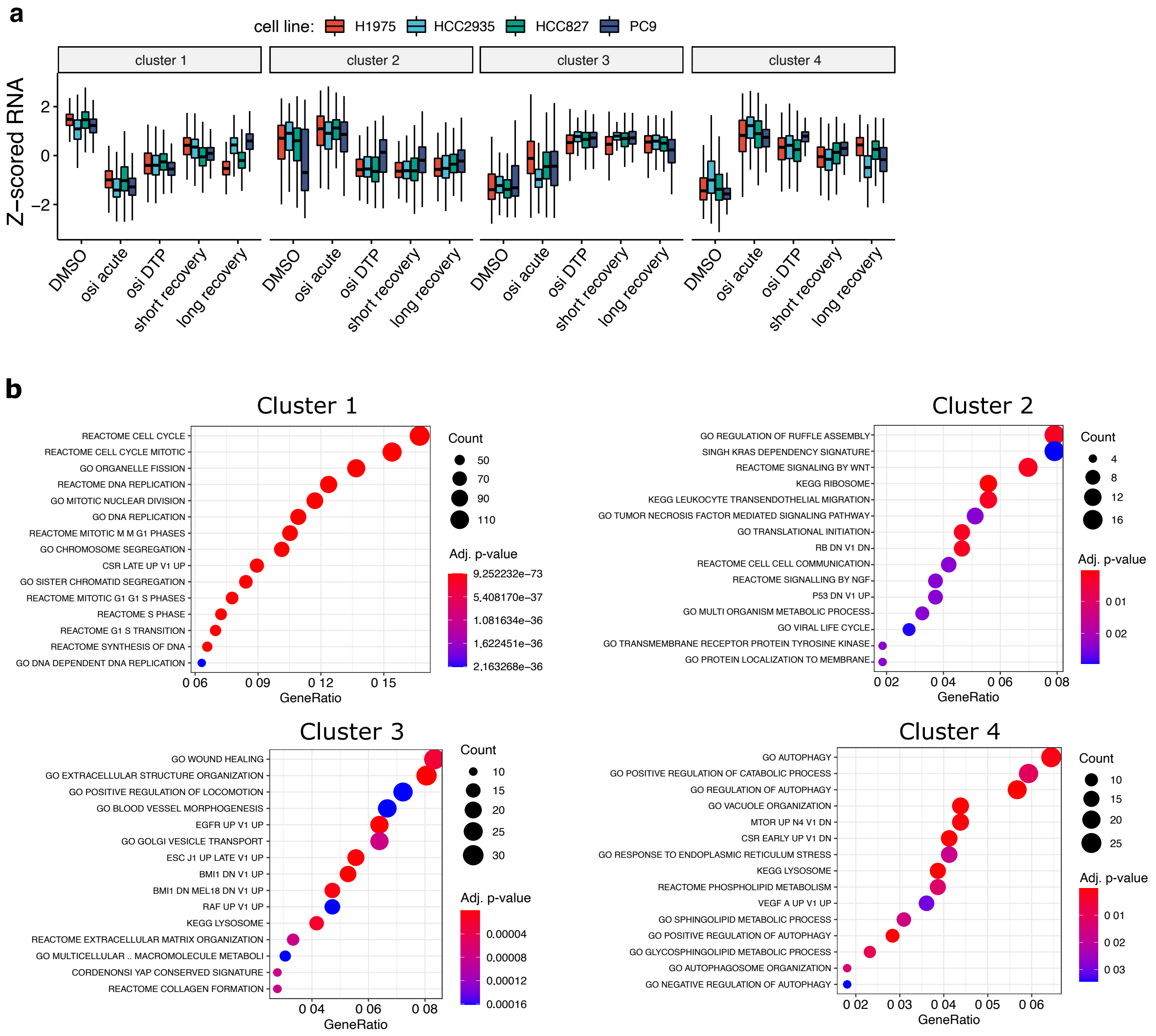

Supplementary Figure 1. RNA expression patterns of osimertinib DTPs and acute treatment. A) RNA expression patterns of clusters identified in Fig. 1A using a moderated F-statistic for the top 2000 genes (ranked by FDR) identified to change in any experimental comparison. Boxplot is quartiles with range bar as minimum or maximum value within 1.5 times the interquartile range. **B)** Pathway enrichment using a hypergeometric test performed on the four gene expression clusters identified in Fig. 1A.

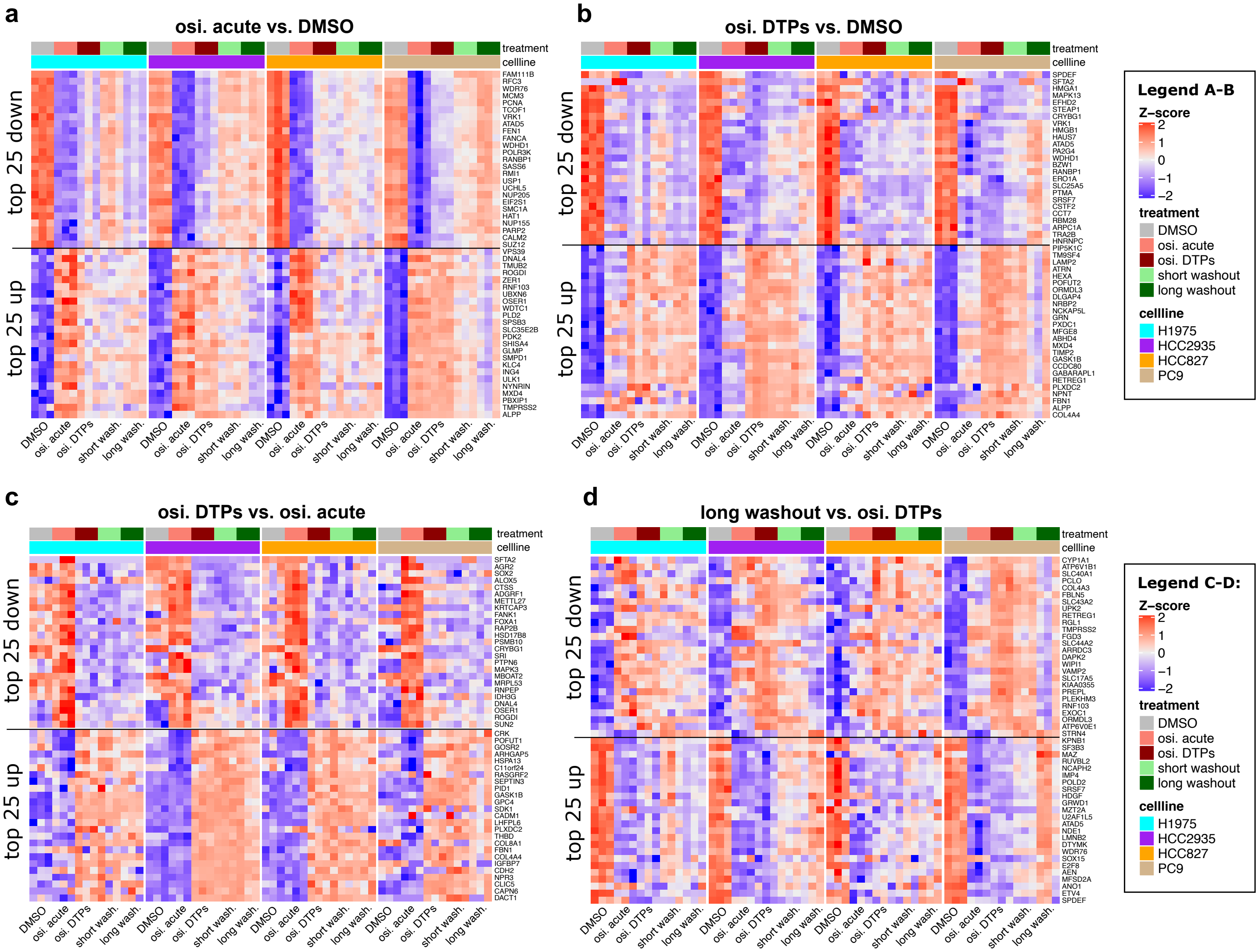

Supplementary Figure 2. Top differentially expressed genes in osimertinib DTPs and acute treatment. A-D) Heatmaps of top ranked (selected by lowest FDR of covariate analysis) gene expression changes from four differential gene expression comparisons controlling for cell line covariate. Genes are Z-scored by cell line using normalized log2TPM expression values and top 25 up and top 25 down genes are displayed for each comparison. **A)** osimertinib acute vs. DMSO top genes **B)** osimertinib DTPs vs. DMSO top genes **C)** osimertinib DTPs vs. osimertinib acute top genes **D)** long washout vs. osimertinib DTPs top genes.

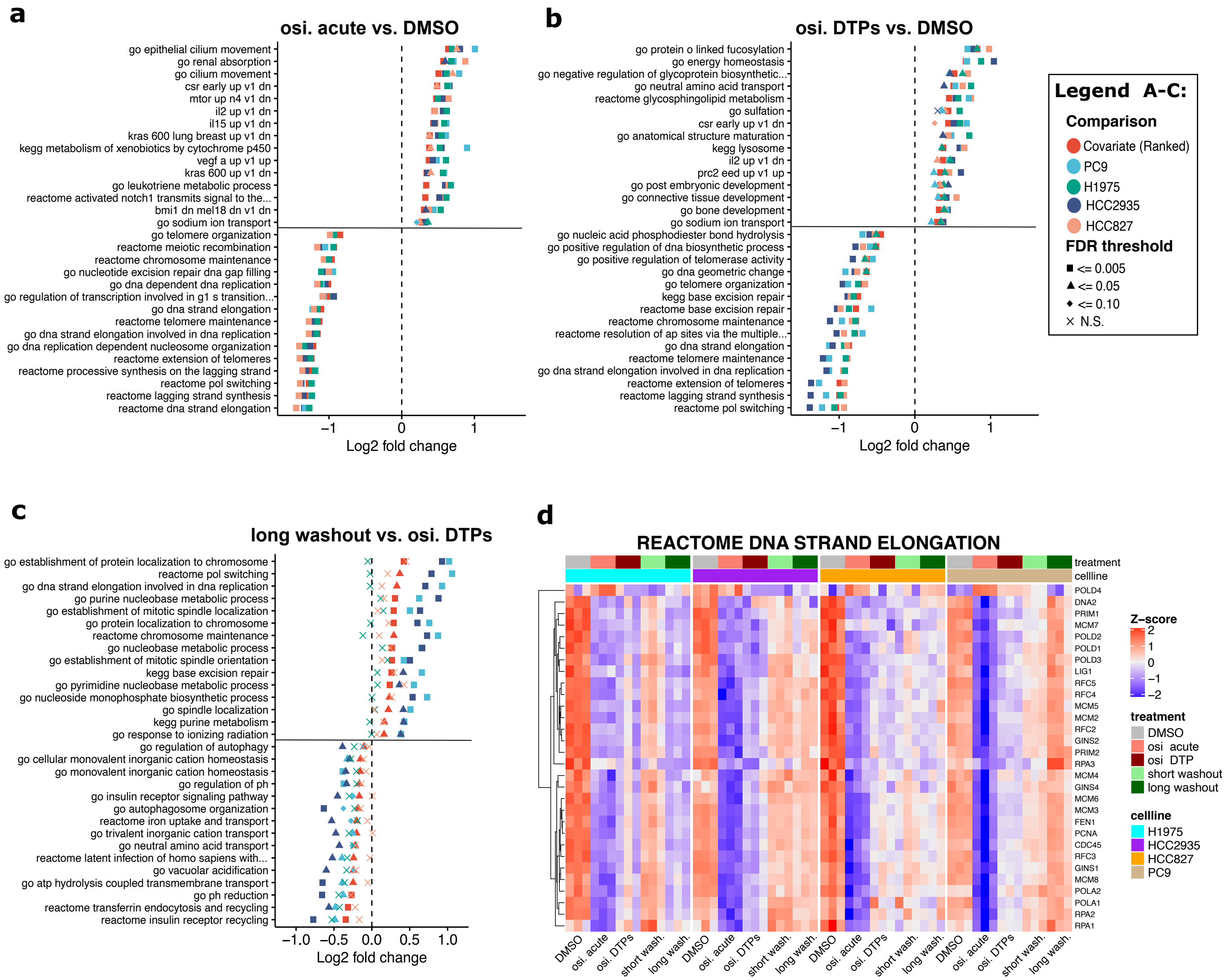

Supplementary Figure 3. Top differentially expressed pathways in osimertinib DTPs and acute treatment. A-C) Top ranked gene expression pathway changes, selected by lowest FDR of cell line covariate differential GSVA analysis, compared to comparisons done separately in each cell line. The pathways are ordered by cell line covariate log2 fold change, color indicates the specific comparison, and shape indicates FDR status. **A)** osimertinib acute vs. DMSO top pathways **B)** osimertinib DTPs vs. DMSO top pathways **C)** long washout vs. osimertinib DTPs top pathways **D)** Example of cell-cycle related Reactome DNA strand elongation pathway identified to decrease in osimertinib acute treatment and DTPs versus DMSO. Gene expression log2TPM values are Z-scored normalized by cell line.

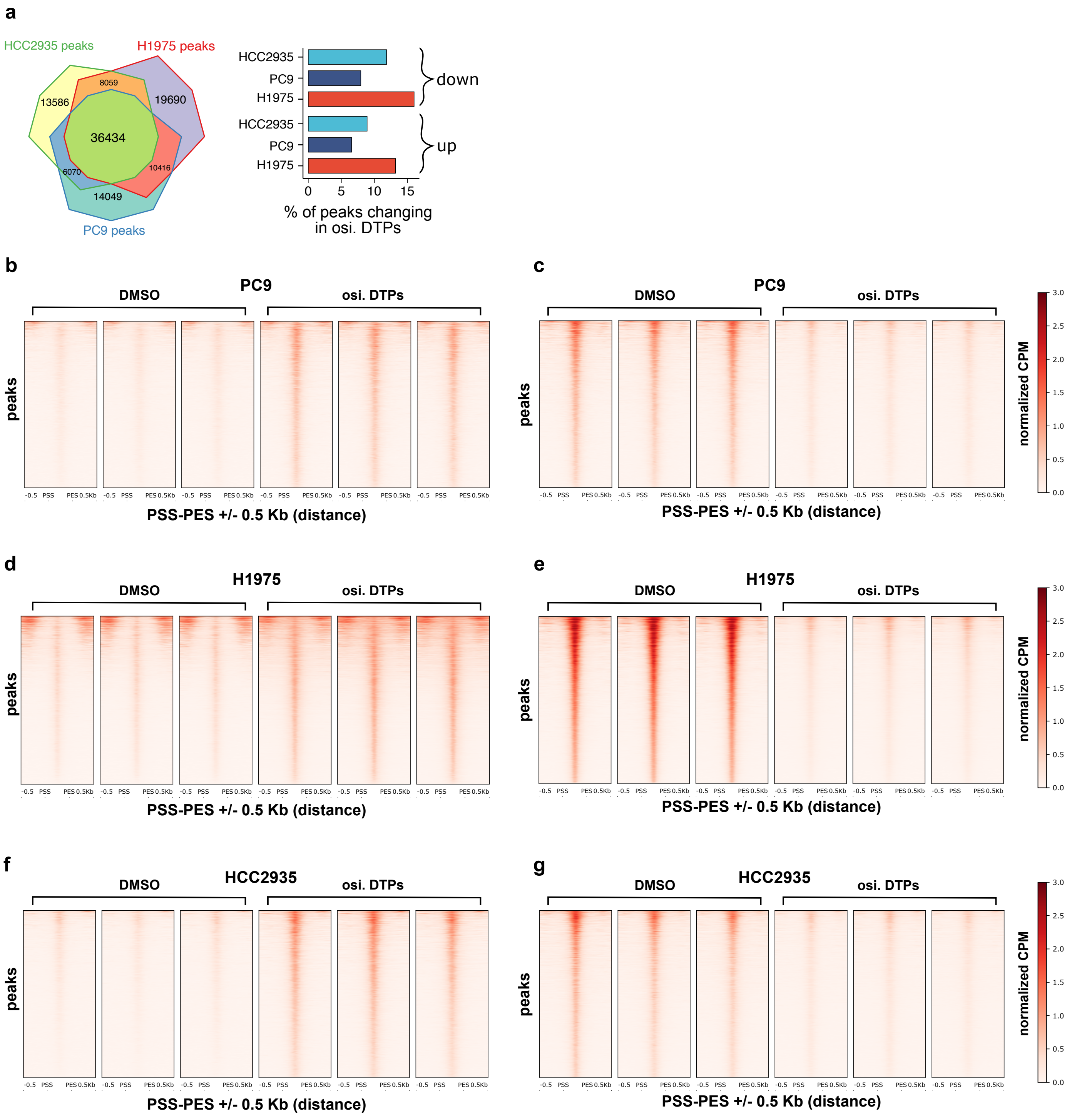

Supplementary Figure 4. Osimertinib DTPs display chromatin accessibility alterations across EGFR mutant cell line models. A) Left Panel: Overlap of high confidence ATAC-seq consensus peaks from each cell line. **Right panel:** Percentage of consensus peaks identified to change significantly up or down in each cell line (at least two-fold change and FDR < 0.005). **B)** Peaks identified to gain accessibility in PC9 osimertinib DTPs by differential peak analysis (> 2-fold change and FDR < 0.005). Peaks are normalized counts per million centered on peak center. Each peak is 500 bp from peak start site (PSS) to peak end site (PES) +/- 0.5 kilobase-pairs. **C)** PC9 osimertinib DTPs down peaks (< -2-fold change and FDR < 0.005). **D)** H1975 osimertinib DTPs up peaks, same as B. **E)** H1975 osimertinib DTPs down peaks, same as C. **F)** HCC2935 osimertinib DTPs up peaks, same as B. **G)** HCC2935 osimertinib DTPs down peaks, same as C.

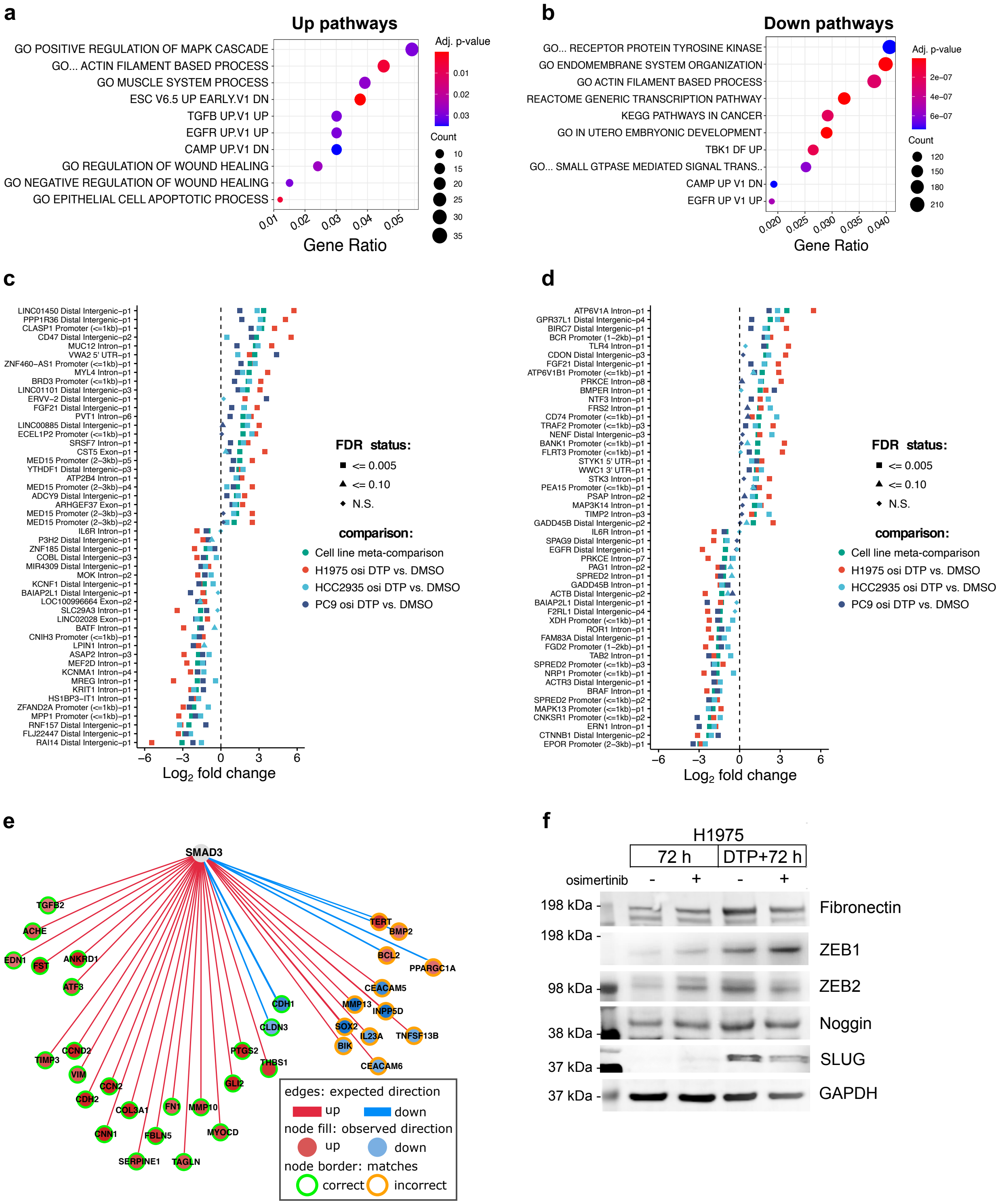

Supplementary Figure 5. Osimertinib DTPs display gene regulatory changes in receptor tyrosine kinase pathway, EMT-related pathway, and SMAD signaling genes. A) Pathways enriched for increased chromatin accessibility regions in DTPs from ATAC-seq meta-analysis across cell lines using a hypergeometric test. **B)** Pathways enriched for decreased chromatin accessibility regions in DTPs. **C)** Top-ranked changing peaks in DTPs from ATAC-seq meta-analysis across cell lines (selected by lowest FDR) compared to behavior in individual cell lines. The peaks are ordered by cell line meta-comparison average log2 fold change, color indicates the specific comparison, and shape indicates FDR status. **D)** Top-ranked changing peaks in DTPs for genes overlapping MAPK-related pathways, same as C. **E)** SMAD3 was inferred to have increased transcription factor activity in osimertinib DTPs (Pollard p-value=7.31E-09) by Causal Reasoning analysis (using the cell line covariate comparison). The edge color shows expected direction, node fill shows observed direction, and the node outline displays whether expected direction matches the observed direction. **F)** Western blot of EMT-related proteins and transcription factors in H1975 treated with osimertinib for 72 hrs. or 3 weeks to form DTPs with or without 72 hr. washout.

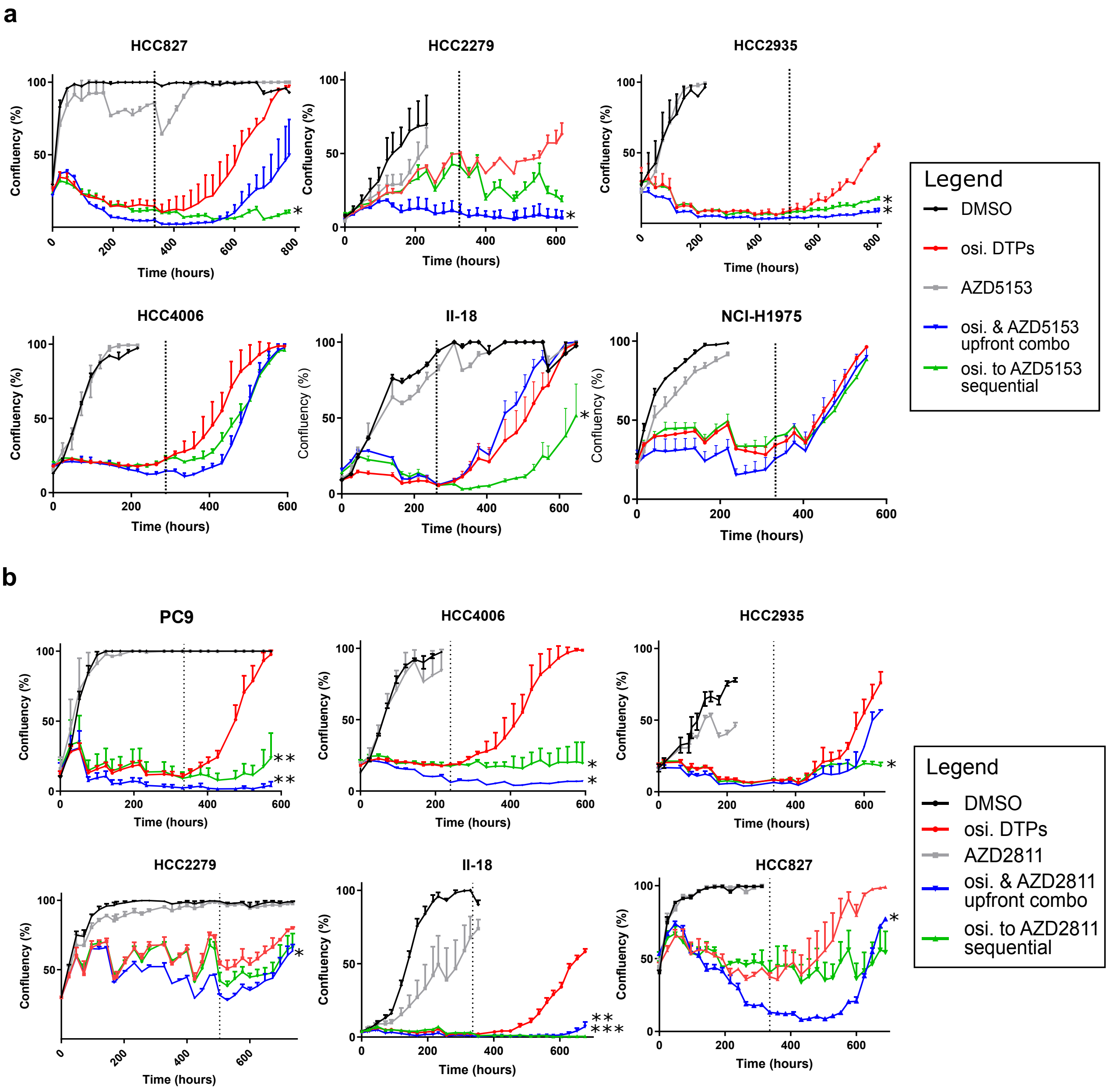

Supplementary Figure 6. The BRD4 inhibitor AZD5153 and the AURKB inhibitor AZD2811 displayed combination benefit with osimertinib. A) Cell confluency for the osimertinib DTP screen and the BRD4 inhibitor AZD5153. Cells were treated with DMSO control, AZD5153 (300 nM for all cell lines except II-18 and HCC2279 which were dosed at 30 nM), or osimertinib (500 nM) in an upfront combination, or osimertinib followed by AZD5153 dosed sequentially. Dotted lines indicate washout in upfront combination or drug crossover in sequential combination and error is SEM. The significance is a two-sided t-test comparing the individual replicate drug combination AUC versus osimertinib monotherapy control (*p-value* *<0.05, **<0.005, ***<0.001). **B)** Same as A, for the AURKB inhibitor AZD2811 (dosed at 100 nM).

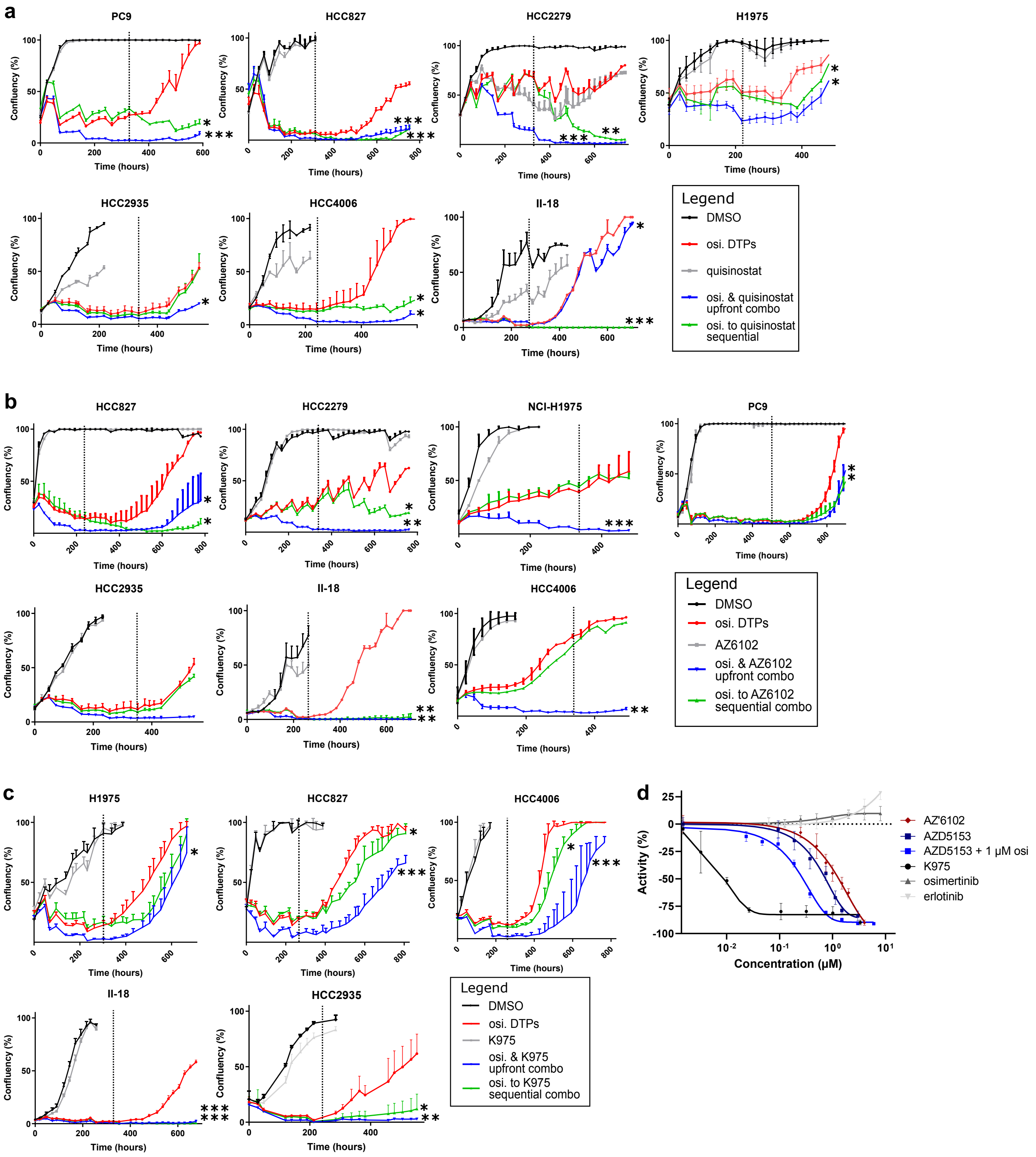

Supplementary Figure 7. The HDAC inhibitor quisinostat, the TNKS inhibitor AZ6102, and the TEAD inhibitor K-975 displayed combination benefit with osimertinib. A) Cell confluency for the osimertinib DTP screen and the HDAC inhibitor quisinostat. Cells were treated with DMSO control, quisinostat (10 nM), or osimertinib (500 nM) and quisinostat in an upfront combination, or osimertinib followed by quisinostat dosed sequentially. Dotted lines indicate washout in upfront combination or drug crossover in sequential combination and error is SEM. The significance is a two-sided t-test comparing the individual replicate drug combination AUC versus osimertinib monotherapy control (*p-value* *<0.05, **<0.005, ***<0.001). **B)** Same as A, for the TNKS inhibitor AZ6102 (dosed at 1 µM). **C)** Same as A, for the TEAD inhibitor K975 (dosed at 100 nM). **D)** Percent activity of TEAD luciferase in a TEAD reporter assay using a 10-point dose-response of osimertinib, erlotinib, K975, AZD5153, AZ6102 or AZD5153 in combination with osimertinib.

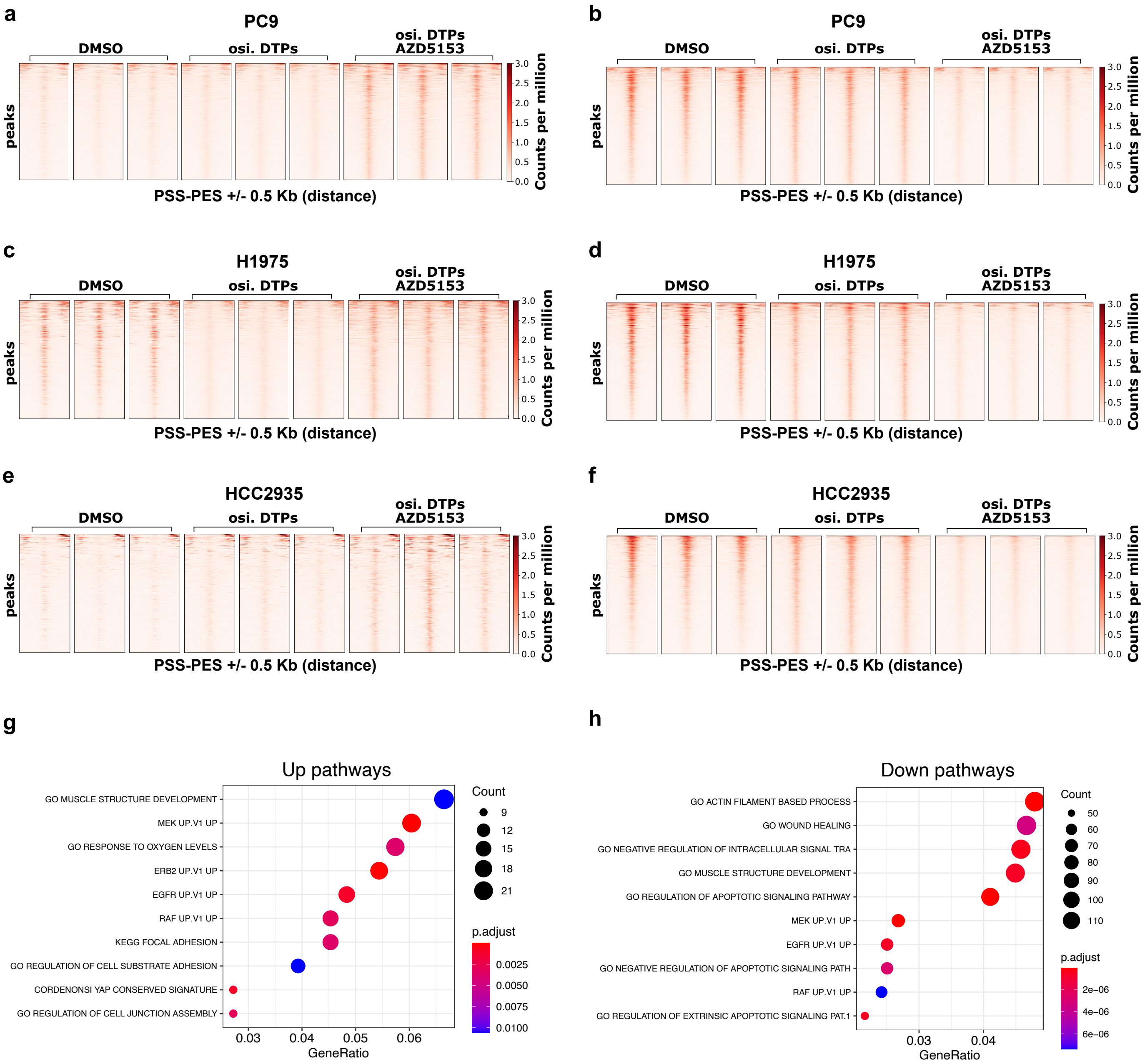

Supplementary Figure 8. Osimertinib DTPs treated with the BRD4 inhibitor AZD5153 show further chromatin accessibility changes. A) ATAC-seq peaks identified to gain accessibility in PC9 BRD4 inhibitor AZD5153 treated osimertinib DTPs versus DTPs without AZD5153 (> 2-fold change and FDR < 0.005). Peaks are normalized counts per million centered on peak center. Each peak is 500 bp from peak start site (PSS) to peak end site (PES) +/- 0.5 kilobase-pairs. **B)** Decreased accessibility peaks in AZD5153-treated osimertinib DTPs versus DTPs without AZD5153 (< -2-fold change and FDR < 0.005). **C)** Same as A, for H1975 cell line. **D)** Same as B, for H1975 cell line. **E)** Same as A, for HCC2935 cell line. **F)** Same as B, for HCC2935 cell line. **G)** Pathways enriched for increased chromatin accessibility genes in AZD5153 treated osimertinib DTPs versus DTPs without AZD5153 from ATAC-seq meta-analysis across cell lines using a hypergeometric test. **H)** Pathways enriched for decreased chromatin accessibility genes in AZD5153 treated osimertinib DTPs versus DTPs without AZD5153.

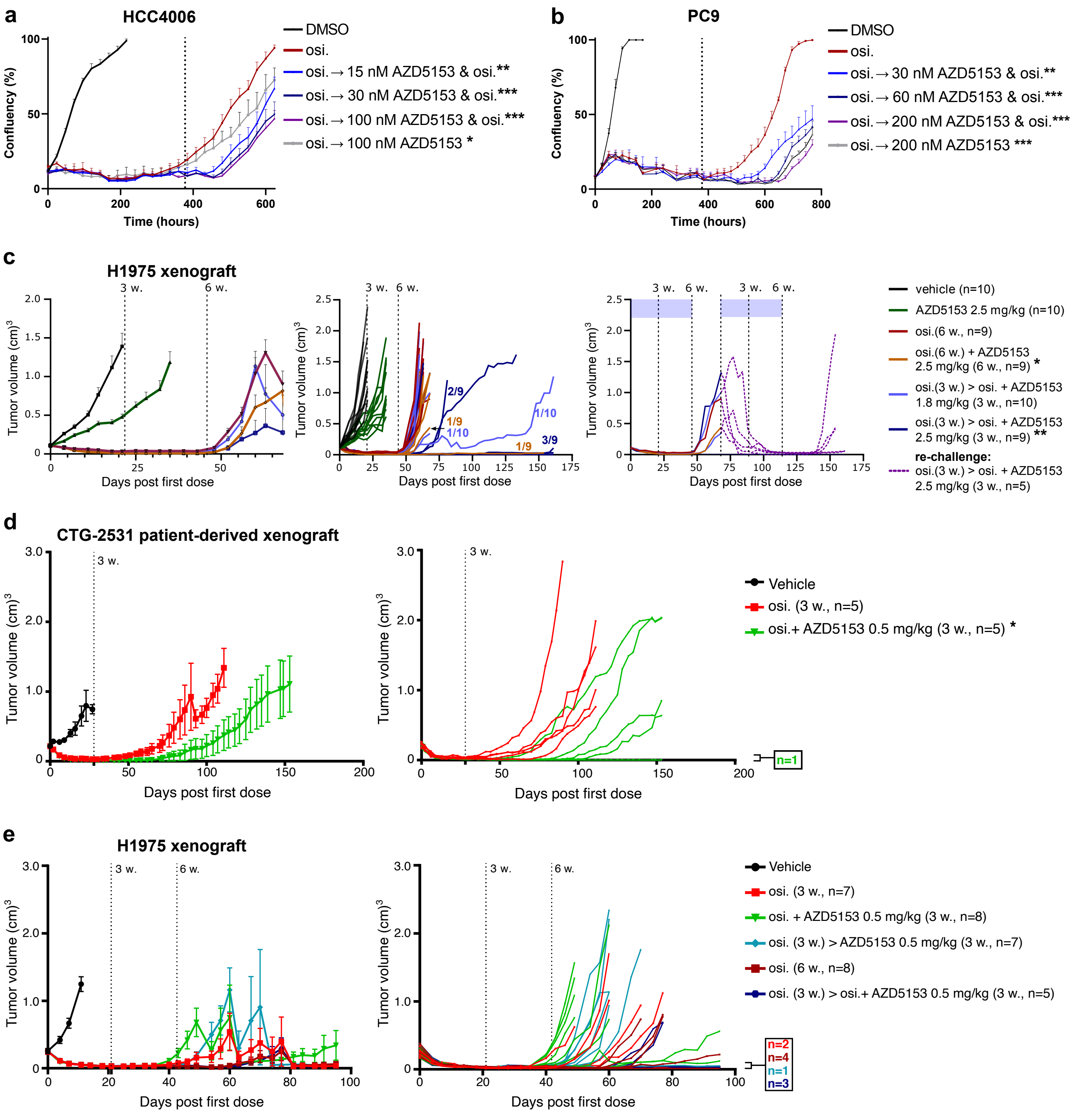

Supplementary Figure 9. The combination benefit observed for the BRD4 inhibitor AZD5153 with osimertinib is dose dependent. **A)** HCC4006 cells were treated with osimertinib to obtain DTPs and switched to 15 nM, 30 nM, or 100 nM AZD5153 in combination with osimertinib or 100 nM AZD5153 monotherapy. For comparison 300 nM AZD5153 monotherapy was used in the drug screens. Dotted lines indicate drug crossover in sequential combination. Error is s.e.m. and the legend includes significance from a two-sided t-test versus osimertinib monotherapy end point (*p-value* *<0.05, **<0.005, ***<0.001). **B)** Same as A, for PC9 cells dosed with a switch to 30 nM, 60 nM, or 200 nM AZD5153 in combination with osimertinib or 200 nM AZD5153 monotherapy. **C)** A H1975 xenograft tumor regrowth study where tumor volume was monitored following osimertinib monotherapy or multiple 1.8 or 2.5 mg/kg combination regimens with AZD5153. At day 68 of the study some tumors were re-randomized and rechallenged with a second AZD5153 combination regimen. **Left:** Average tumor volume up until day 68 when a subset of tumors was re-randomized. Error is s.e.m. and the legend significance is from a two-sided t-test versus osimertinib monotherapy control at 63 days (*p-value* *<0.05, **<0.005, ***<0.001). **Center:** Individual tumor responses without drug re-challenge measurements. **Right:** Individual tumor responses of the subset of tumors re-randomized and re-challenged with a second round of AZD5153 combination regimen. **D)** A CTG-2531 (EGFR exon 19 deleted) patient derived xenograft tumor regrowth study where tumor volume was monitored following osimertinib monotherapy or osimertinib in combination with 0.5 mg/kg AZD5153. Same as C right and center panels. **E)** A H1975 xenograft tumor regrowth study where tumor volume was monitored following osimertinib monotherapy or multiple combination regimens with 0.5 mg/kg AZD5153. Same as C, no drug combinations were significant in H1975.

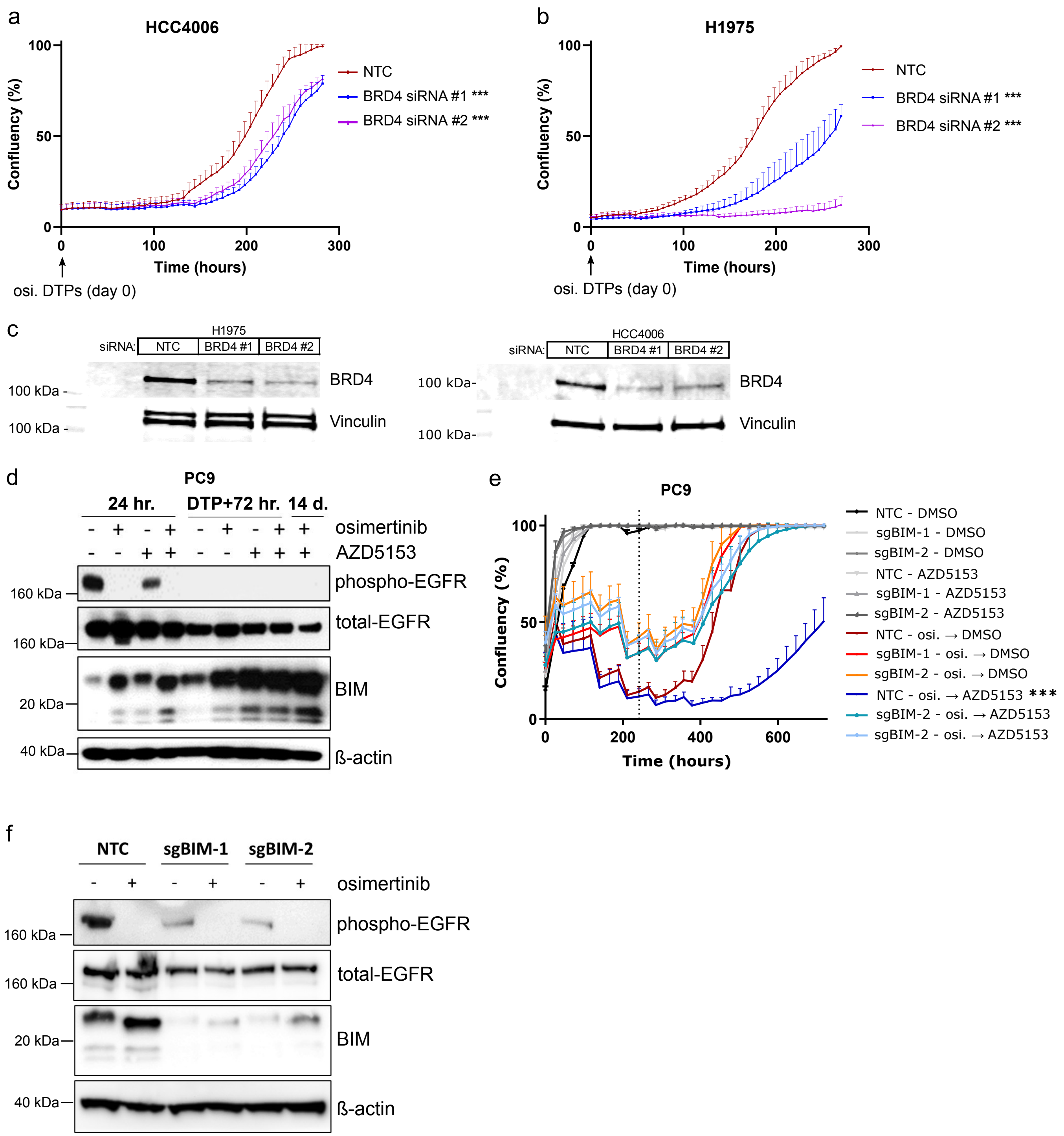

Supplementary Figure 10. The vulnerability of osimertinib DTPs to AZD5153 is driven by the direct target BRD4 and is dependent on BIM upregulation. A) Cell confluency of HC4006 cells treated with osimertinib to obtain DTPs, replated, and transfected with BRD4 siRNAs or NTC control. In this experiment day 0 cells are replated DTPs transfected with BRD4 siRNA unlike other confluency experiments where day 0 is before DTPs are generated. Error is s.e.m and the legend includes significance from a two-sided t-test versus osimertinib monotherapy NTC endpoint (*p-value* *<0.05, **<0.005, ***<0.001). **B)** Same as A, in H1975 cell line. **C)** Western blot validation of BRD4 knockdown efficiency of siRNA #1 and #2. **D)** Western blot of BIM protein in PC9 cells treated with osimertinib for 24 hr. or in DTPs treated with and without AZD5153 for an additional 72 hrs. or 14 days. **E)** BIM knockout using two CRISPR guides in osimertinib PC9 DTPs treated sequentially with AZD5153 (300 nM) or a DMSO control. Dotted lines indicate drug crossover in sequential combination and error is s.e.m. and the legend includes significance from a two-sided t-test versus osimertinib monotherapy NTC endpoint (*p-value* *<0.05, **<0.005, ***<0.001). **F)** Validation of BIM knockout CRISPR guides by Western blot in PC9 cells.

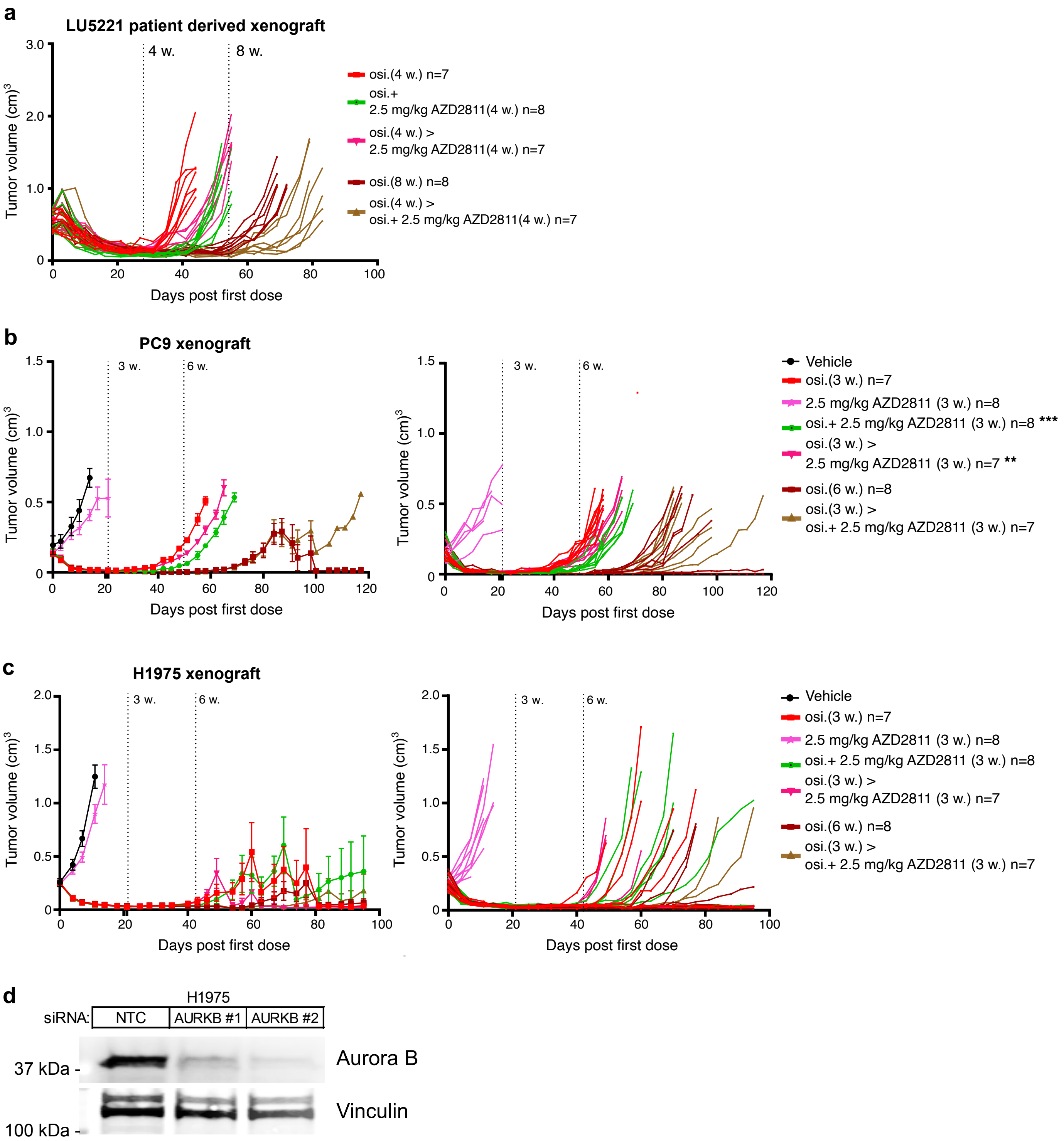

Supplementary Figure 11. The AURKB inhibitor AZD2811 displays combination benefit with osimertinib in two of three models examined in vivo. A) Individual tumor regrowth patterns from a LU5221 EGFR exon 19-deleted patient derived xenograft tumor regrowth study where tumor volume was monitored following osimertinib monotherapy or multiple regimens with AZD2811 (dosed IV 25mg/kg once weekly). **B)** A PC9 xenograft tumor regrowth study where tumor volume was monitored following osimertinib monotherapy or multiple regimens with AZD2811 (dosed IV 25mg/kg once weekly). **Left:** Average tumor volume. Error is s.e.m. and the legend significance is from a two-sided t-test versus osimertinib monotherapy control at 58 days (*p-value* *<0.05, **<0.005, ***<0.001). **Right:** Individual tumor responses **C)** Same as B, using a H1975 tumor xenograft model. . None of the AZD2811 combinations in H1975 were significant. **D)** Validation of AURKB siRNA knockdown by Western blot.

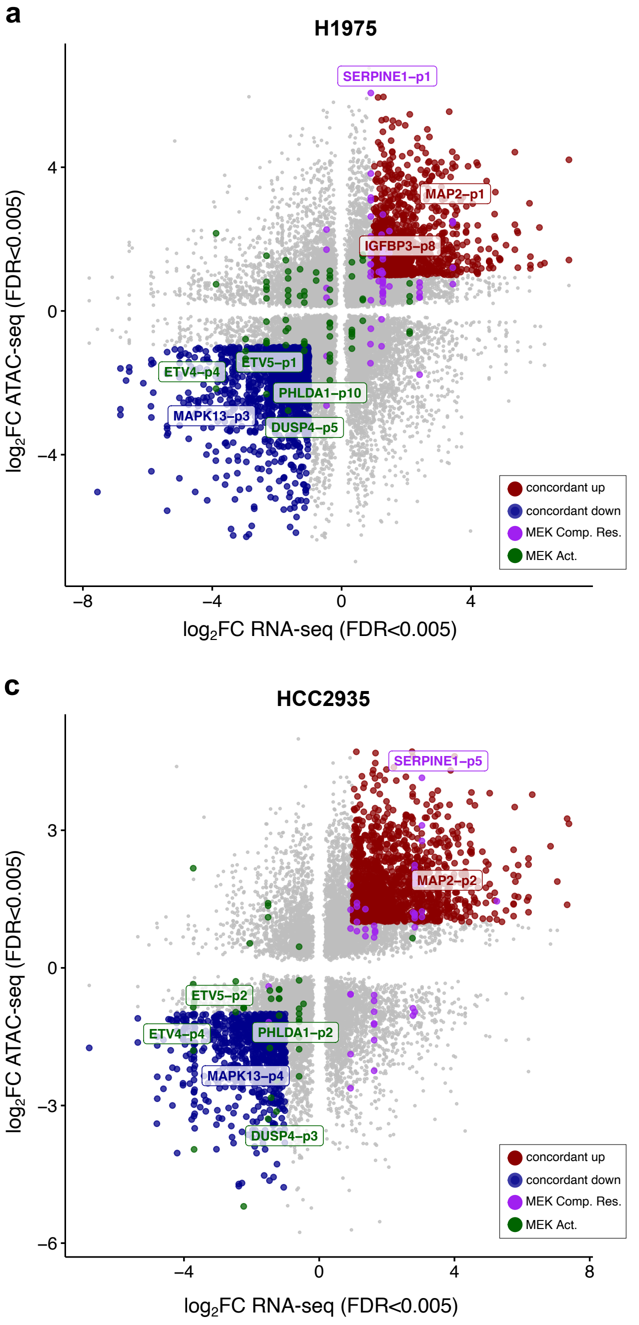

Supplementary Figure 12. Osimertinib DTPs display concordant chromatin accessibility and gene expression changes across many genes. A) Comparison of log2FC gene expression changes versus peak log2FC that were significantly changed in RNA expression (FDR<0.005) and chromatin accessibility (FDR<0.005) in H1975 osimertinib DTPs versus DMSO. Concordant up genes (dark red) change at least two-fold up in ATAC-seq and RNA-seq, concordant down genes (dark blue) do the opposite. MEK activation genes are dark green and MEK compensatory resistance genes are purple. Labeled genes correspond to genome browser examples in Fig. 2C, Fig. 5D, and Supplementary Fig. 13 A-F. **B)** Same as A, for PC9. **C)** Same as A, for HCC2935.

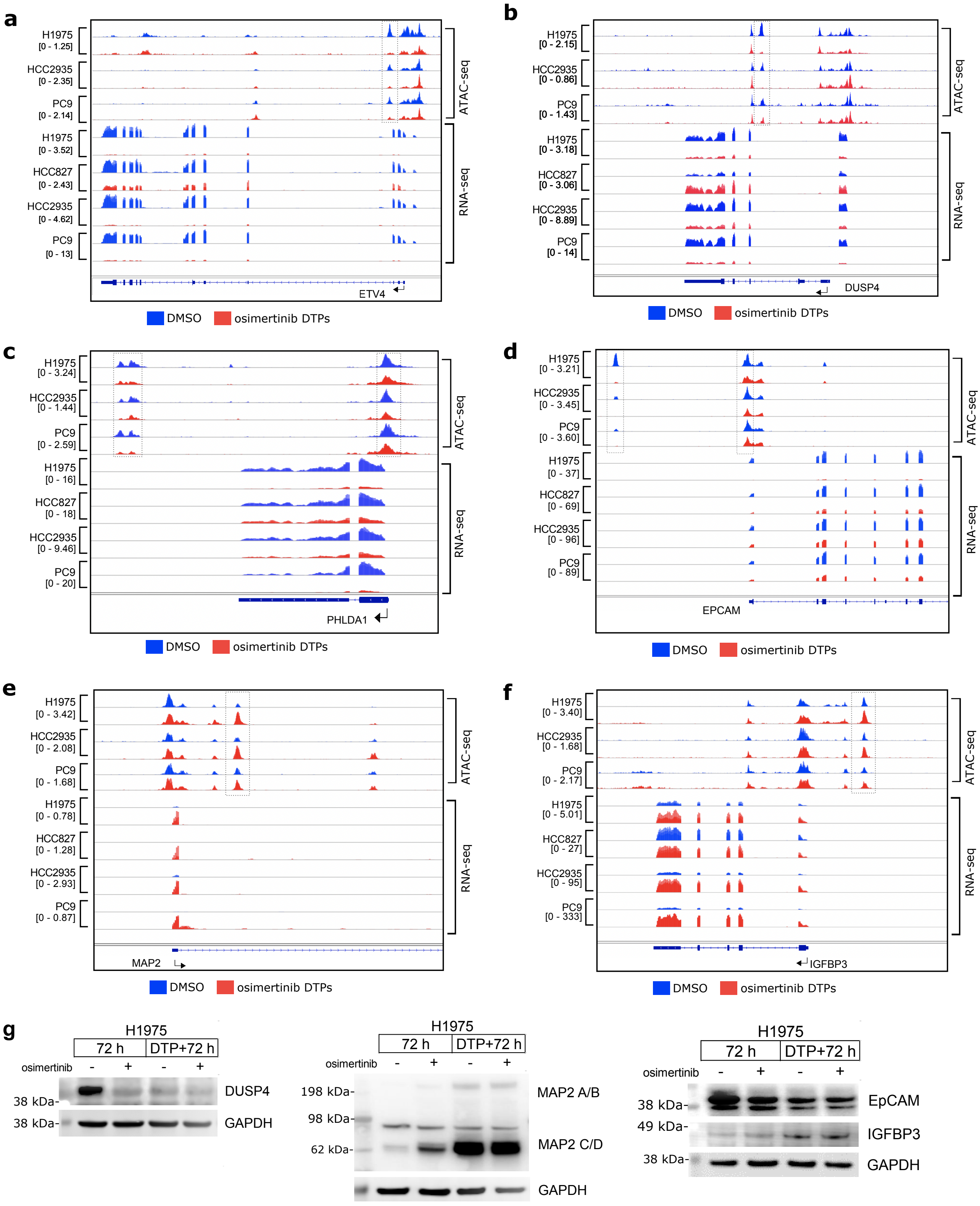

Supplementary Figure 13. Example genes showing concordant chromatin accessibility and gene expression changes in osimertinib DTPs. A-F) Genome browser views of gene signal in normalized counts per million that were identified to change chromatin accessibility and RNA expression in osimertinib DTPs versus DMSO. Each comparison of treatment (osimertinib DTPs) versus control (DMSO) was group scaled to the same normalized counts per million range to enable comparison. Genome browser views of **A)** ETV4 decreasing, **B)** DUSP4 decreasing, **C)** PHLDA1 decreasing, **D)** EPCAM decreasing, **E)** MAP2 increasing, and **F)** IGFBP3 increasing in osimertinib DTPs. **G) Left:** Western blot of DUSP4 protein in H1975 treated with osimertinib for 72 hrs. or 3 weeks to form DTPs with or without 72 hr. washout. **Center:** Western blot of MAP2 protein levels. **Right:** Western blot of EpCAM and IGFBP3 protein levels.

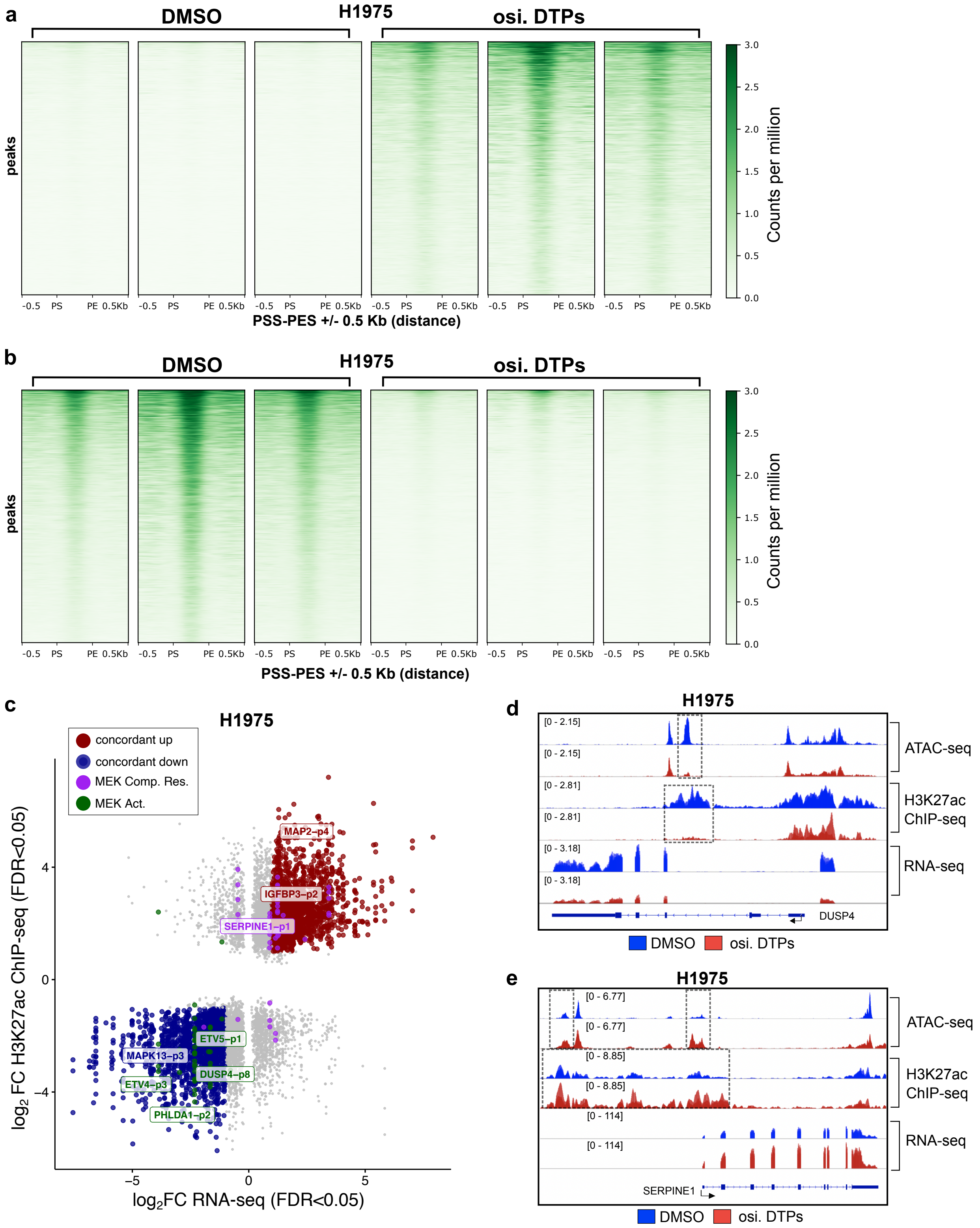

Supplementary Figure 14. The H3K27ac changes in osimertinib DTPs are concordant with ATAC-seq chromatin accessibility changes. A) H3K27ac peaks identified to increase in H1975 osimertinib DTPs (>2-fold change and FDR <0.005). Peaks are normalized counts per million centered on peak center. Each consensus peak is 500 bp from peak start site (PSS) to peak end site (PES) +/- 0.5 kilobase-pairs. **B)** H3K27ac peaks identified to decrease in H1975 osimertinib DTPs (< -2-fold change and FDR <0.005). **C)** Comparison of log2FC gene expression changes versus H3K27ac peak log2 fold changes that were significantly changing in RNA expression (FDR<0.05) and H3K27ac levels (FDR<0.05) in H1975 osimertinib DTPs versus DMSO. Concordant up genes (dark red) change at least two-fold up in ATAC-seq and RNA-seq, concordant down genes (dark blue) do the opposite. MEK activation genes are dark green and MEK compensatory resistance genes are purple. Labeled genes correspond to genome browser examples in Fig. 2C, Fig. 5D, and Supplementary Fig. 13 A-F. **D)** Genome browser view of DUSP4 identified to change chromatin accessibility, H3K27ac levels, and RNA expression in H1975 osimertinib DTPs versus DMSO. Each comparison of osimertinib DTPs versus control (DMSO) was group scaled to the same normalized counts per million range to enable comparison. **E)** Genome browser view of SERPINE1, same as D.

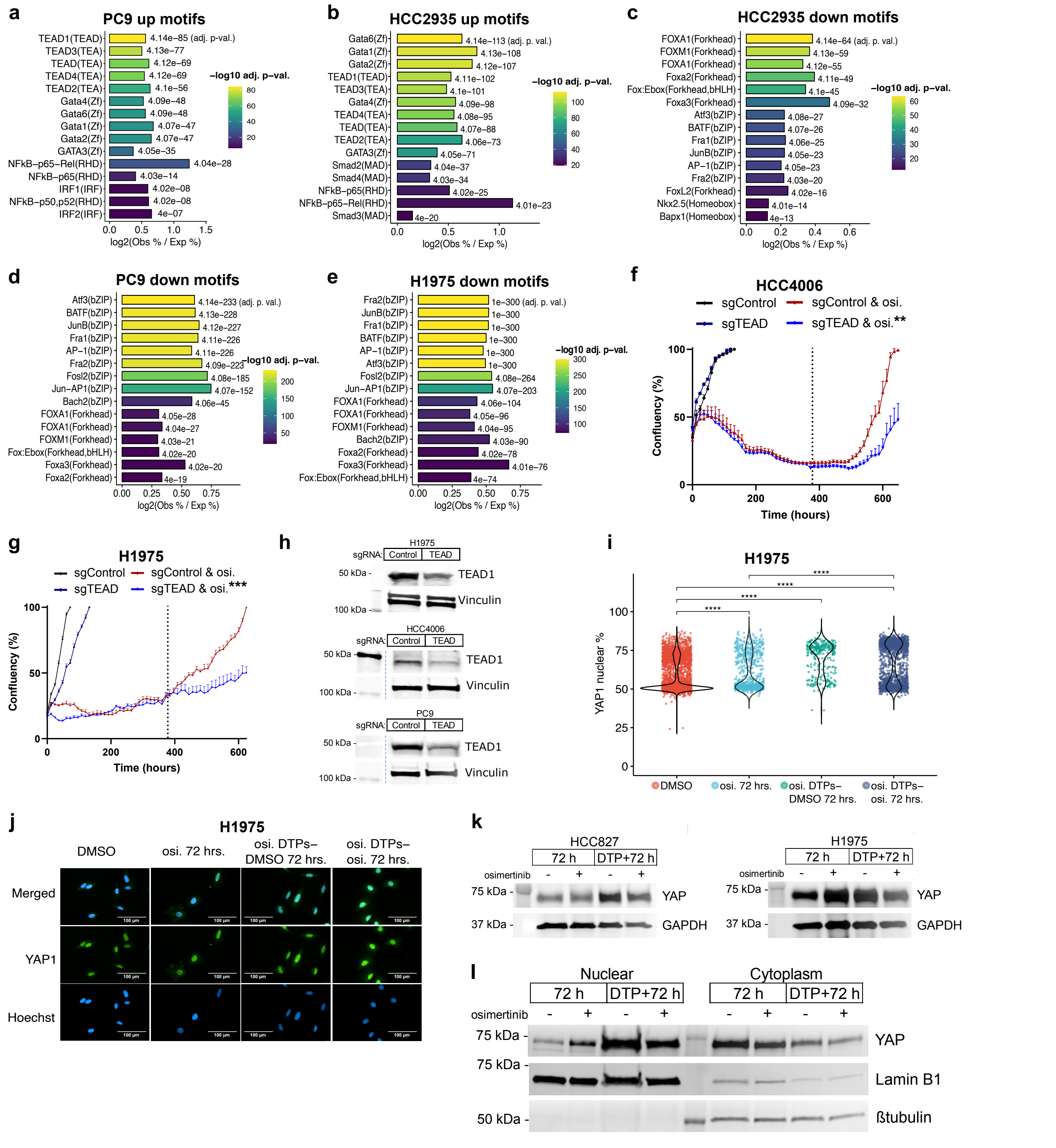

Supplementary Figure 15. Osimertinib DTPs turn off the Hippo pathway and vulnerable to TEAD knockout. A-B) Top known transcription factor motifs enriched for gained accessibility peaks (top 15 by lowest FDR, bar label is FDR value) in **(A)** PC9 and **(B)** HCC22935 osimertinib DTPs. **C-E)** Top known transcription factor motifs enriched for decreased accessibility peaks (top 15 by lowest FDR, bar label is FDR value) in **(C)** HCC2935, **(D)** PC9, **(E)** H1975. **F)** HCC4006 confluency in cells treated with osimertinib combined with upfront knockout of pan-TEAD (CRISPR guide designed against conserved region of TEAD1-4) followed by drug washout (dotted line). Error is s.e.m and the legend includes significance from a two-sided t-test versus osimertinib monotherapy sgControl end point (*p-value**<0.05, **<0.005, ***<0.001). G) Same as F using H1975 cells. **G)** Same as F using H1975 cells. **H)** Knockout efficiency of TEAD1 using pan-TEAD CRISPR reagent in HCC4006, H1975, and PC9 cells. **I)** Quantitation of the percentage YAP nuclear immunofluorescence (YAP nuclear/ total YAP) in single cells for H1975 osimertinib DTPs with wash out (72 hrs.) or continuous treatment (two-sided Wilcoxon signed-rank test, *p-value* ****<0.0001). **J)** Representative images of YAP nuclear immunofluorescence in H1975 osimertinib DTPs with wash out (72 hrs.) or continuous treatment. **K)** Western blot of total YAP protein in H1975 (left panel) or HCC827 (right panel) treated with osimertinib for 72 hrs. or 3 weeks to form DTPs with or without 72 hr. washout. **L)** Subcellular fractionization western blot of nuclear or cytoplasmic fractions of HCC827 treated with osimertinib for 72 hrs. or 3 weeks to form DTPs with or without 72 hr. washout.

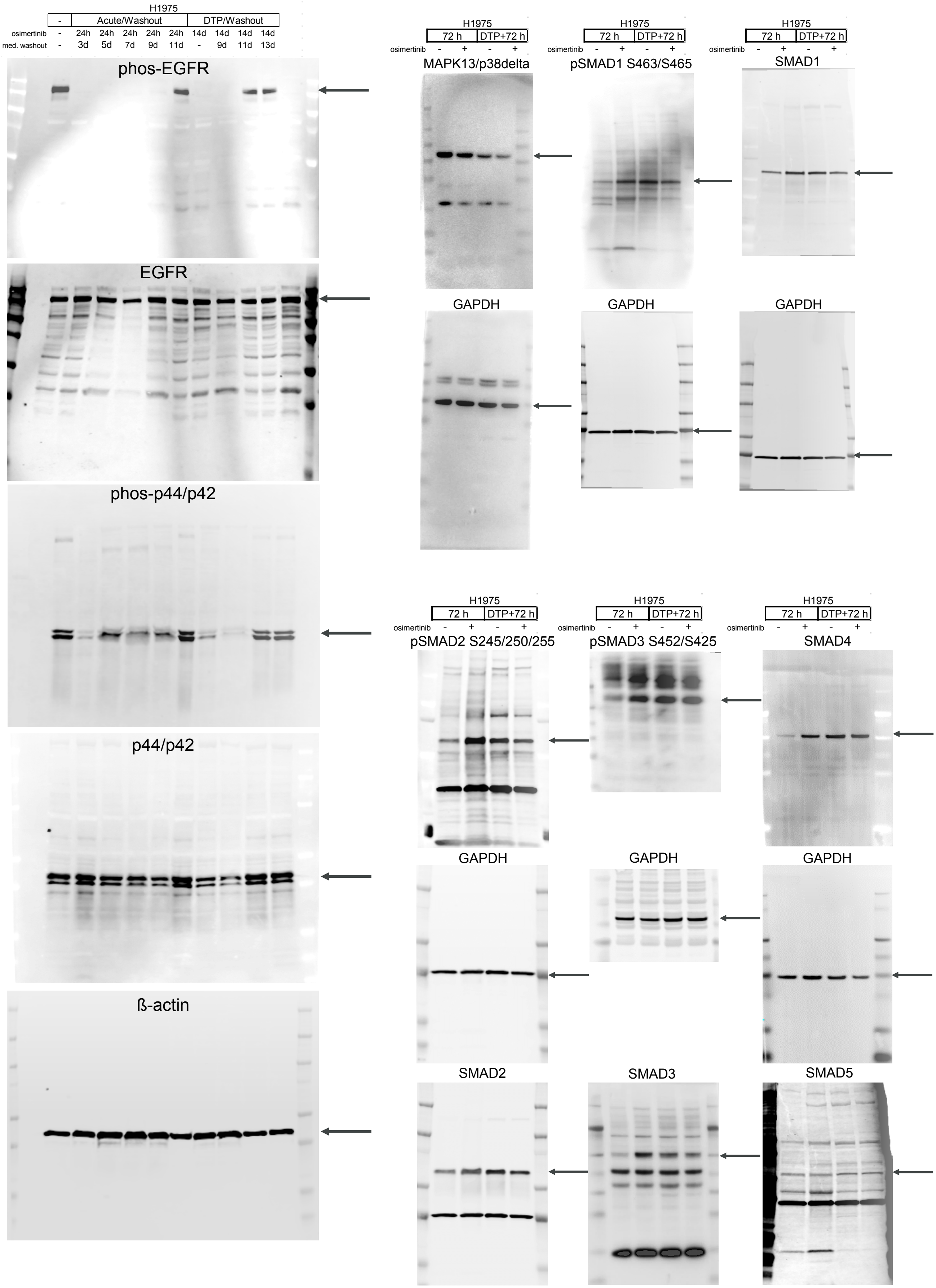

Supplementary Figure 16. Uncropped Western Blots corresponding to Fig. 1B, Fig, 2D, and Fig. 2I.

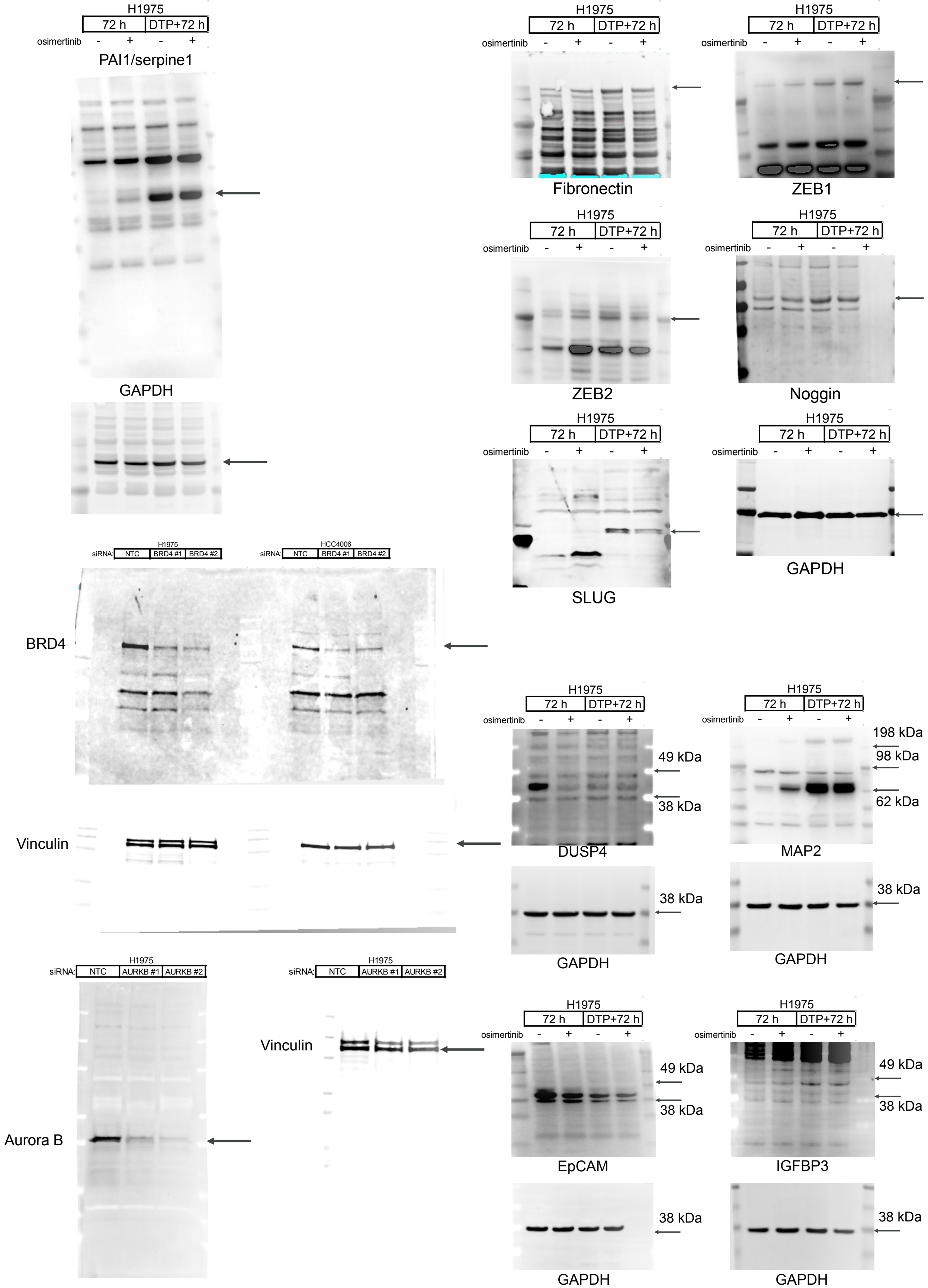

Supplementary Figure 17. Uncropped Western Blots corresponding to Fig. 5E, Supplementary Fig. 5F, Supplementary Fig. 10C, Supplementary Fig. 11D, and Supplementary Fig. 13G.

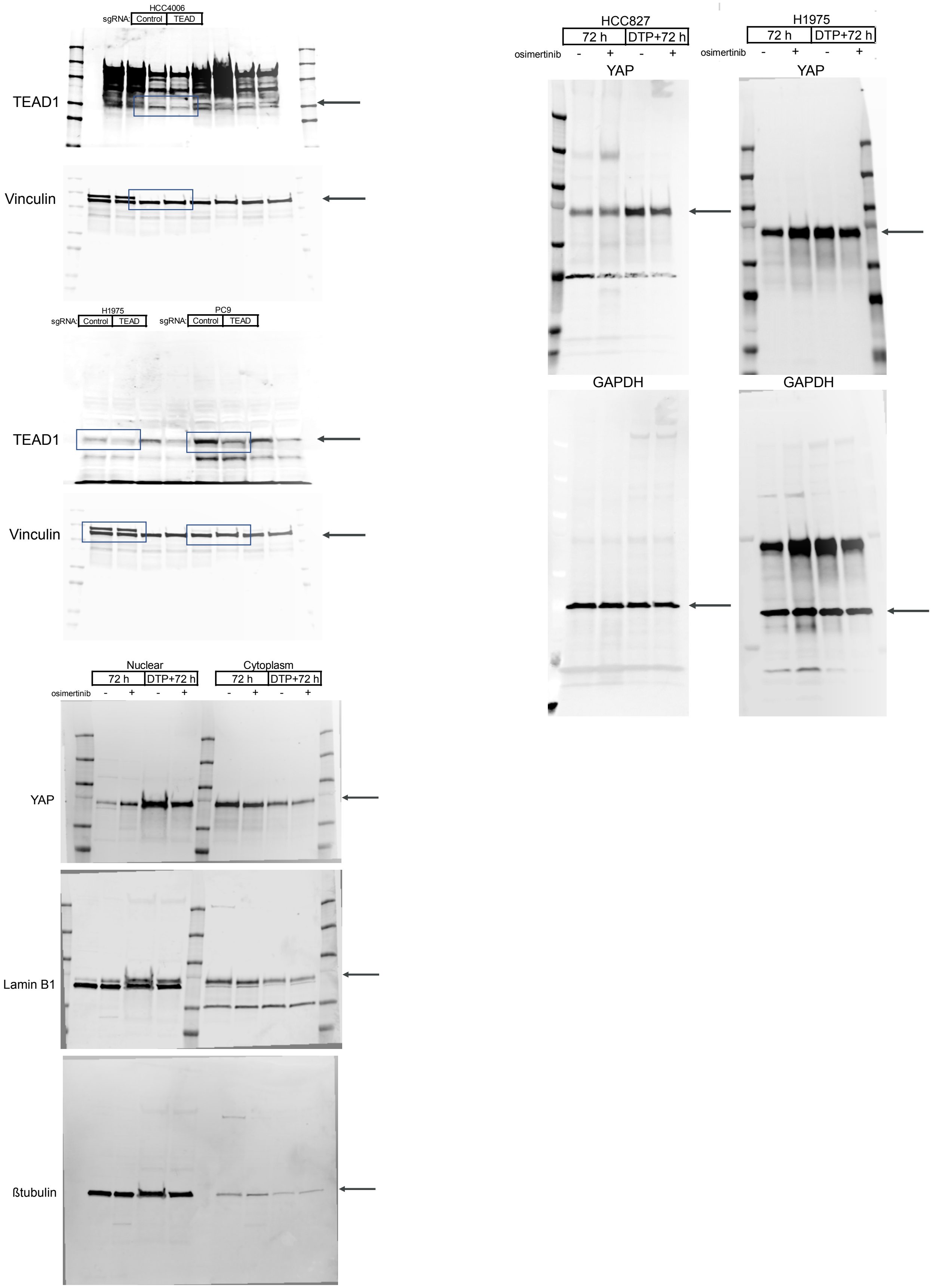

Supplementary Figure 18. Uncropped Western Blots corresponding to Supplementary Fig. 15H, K, and I.

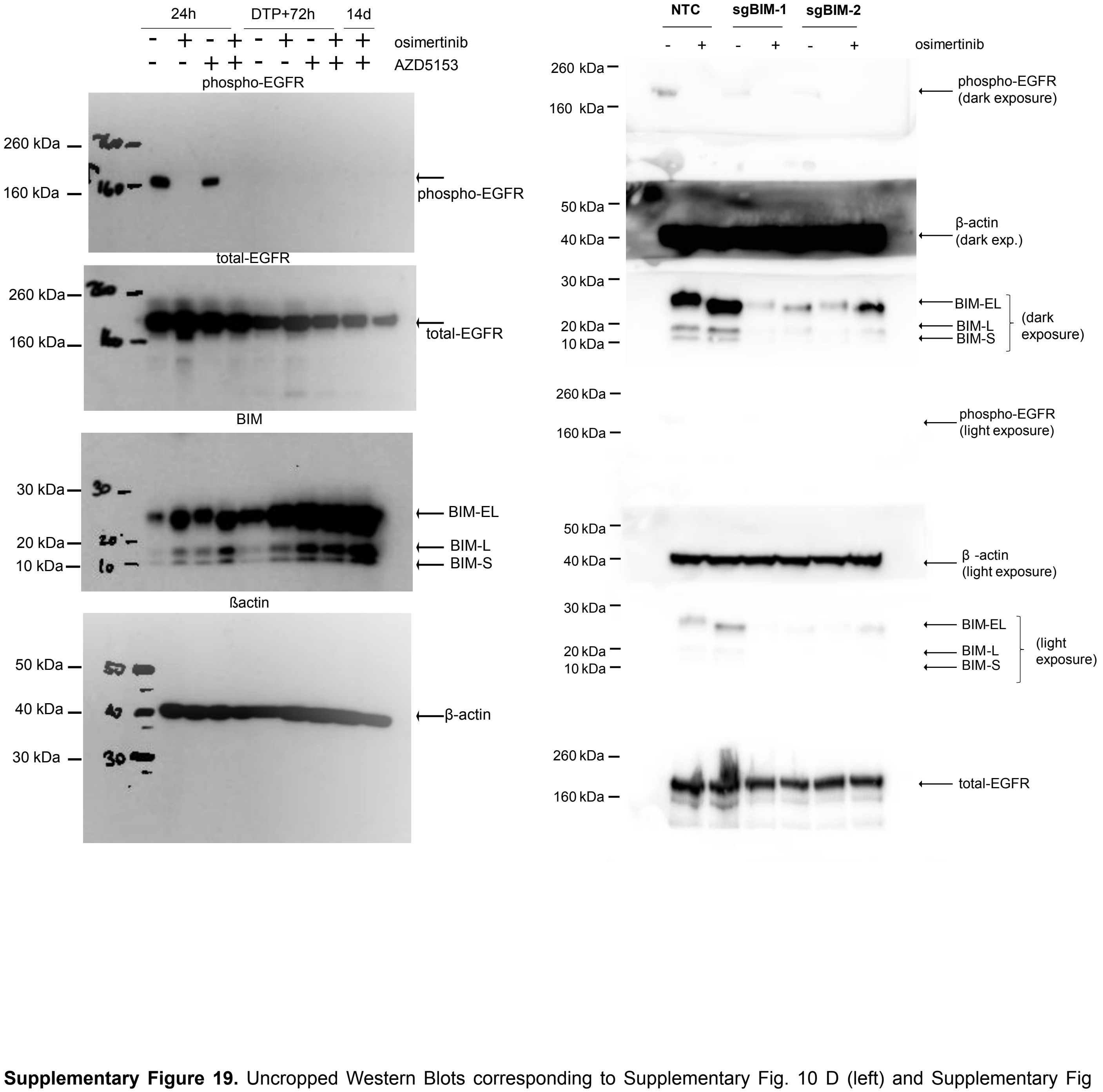

Supplementary Figure 19. Uncropped Western Blots corresponding to Supplementary Fig. 10 D (left) and Supplementary Fig 10F (right). The Western blots for Supplementary Fig. 10D were detected using film and the protein ladder was manually marked by overlay. The Western Blots for Supplementy Fig. 10F were cut into strips for ECL Western Blotting Substrate detection method using chemiluminescence. Molecular weights for Supplementy Fig. 10F are from separate visible spectrum images of blots using Novex Sharp (ThermoFisher) protein ladder.

Supplementary Table 1: RNA-seq alignment and quality control statistics for DMSO controls, osimertinib acute treatment, osimertinib DTPs, and drug washout conditions.

Sample Name	Reads	Aligned Reads	Dup	rRNA	Error rate	% Mapped	5'-3' bias	% GC
H1975 DMSO 1	116.2 M	96.8 M	25.00%	0.50%	0.36%	83.30%	1.11	51%
H1975 DMSO 2	107.7 M	91.9 M	24.70%	0.60%	0.35%	85.30%	1.1	51%
H1975 DMSO 3	109.2 M	92.9 M	24.30%	0.70%	0.35%	85.10%	1.1	51%
H1975 DTP long washout 72h 1	110.1 M	94.6 M	24.70%	1.50%	0.36%	85.90%	1.11	51%
H1975 DTP long washout 72h 2	104.8 M	88.6 M	25.10%	1.30%	0.36%	84.60%	1.12	51%
H1975 DTP long washout 72h 3	109.1 M	93.8 M	25.50%	1.50%	0.36%	86.00%	1.13	52%
H1975 osimertinib acute 1	119.8 M	100.0 M	26.30%	1.40%	0.36%	83.50%	1.12	51%
H1975 osimertinib acute 2	109.7 M	93.4 M	25.10%	1.20%	0.36%	85.20%	1.12	52%
H1975 osimertinib acute 3	103.1 M	87.8 M	24.10%	1.10%	0.36%	85.10%	1.13	51%
H1975 osimertinib DTP 1	113.3 M	95.7 M	25.30%	1.50%	0.36%	84.40%	1.11	51%
H1975 osimertinib DTP 2	125.5 M	105.3 M	25.10%	1.20%	0.35%	83.90%	1.11	50%
H1975 osimertinib DTP 3	107.4 M	92.0 M	25.60%	1.60%	0.36%	85.60%	1.12	51%
H1975 DTP short washout 1	109.9 M	92.6 M	25.30%	1.30%	0.36%	84.20%	1.11	51%
H1975 DTP short washout 2	113.5 M	95.1 M	24.20%	1.10%	0.36%	83.80%	1.15	51%
H1975 DTP short washout 3	117.0 M	98.4 M	25.30%	1.60%	0.36%	84.10%	1.1	51%
HCC2935 DMSO 1	58.0 M	52.6 M	25.50%	0.80%	0.19%	90.60%	1.17	51%
HCC2935 DMSO 2	51.8 M	47.3 M	24.80%	0.70%	0.19%	91.40%	1.15	51%
HCC2935 DMSO 3	51.1 M	46.8 M	25.80%	0.80%	0.19%	91.60%	1.17	50%
HCC2935 DTP long washout 10d 1	61.7 M	56.1 M	25.90%	0.90%	0.19%	91.00%	1.18	51%
HCC2935 DTP long washout 10d 2	55.8 M	50.7 M	26.90%	0.70%	0.18%	90.70%	1.17	51%
HCC2935 DTP long washout 10d 3	50.9 M	47.4 M	25.60%	0.70%	0.20%	93.10%	1.18	52%
HCC2935 osimertinib acute 1	53.6 M	48.9 M	25.30%	1.30%	0.19%	91.30%	1.18	52%
HCC2935 osimertinib acute 2	47.3 M	43.4 M	25.90%	1.40%	0.20%	91.80%	1.21	51%
HCC2935 osimertinib acute 3	51.5 M	48.4 M	25.90%	1.20%	0.21%	94.00%	1.23	51%
HCC2935 osimertinib DTP 1	57.2 M	52.3 M	26.80%	1.00%	0.19%	91.40%	1.17	51%
HCC2935 osimertinib DTP 2	60.4 M	55.1 M	27.00%	1.00%	0.19%	91.30%	1.14	50%
HCC2935 osimertinib DTP 3	50.6 M	46.9 M	25.10%	0.80%	0.19%	92.60%	1.17	51%
HCC2935 DTP short washout 1	54.0 M	49.0 M	25.60%	0.70%	0.19%	90.90%	1.15	51%
HCC2935 DTP short washout 2	53.6 M	47.5 M	26.20%	0.80%	0.18%	88.60%	1.15	51%
HCC2935 DTP short washout 3	50.2 M	46.6 M	26.40%	0.80%	0.20%	92.80%	1.2	51%
HCC827 DMSO 1	49.7 M	45.7 M	26.20%	1.00%	0.21%	92.00%	1.11	51%
HCC827 DMSO 1	54.7 M	50.9 M	27.00%	0.60%	0.18%	93.20%	1.09	50%
HCC827 DMSO 3	57.7 M	53.3 M	27.00%	1.40%	0.18%	92.30%	1.1	50%
HCC827 DTP long washout 96h 1	47.5 M	43.4 M	26.30%	2.70%	0.20%	91.40%	1.09	50%
HCC827 DTP long washout 96h 2	46.2 M	42.1 M	26.20%	2.60%	0.20%	91.20%	1.07	51%
HCC827 DTP long washout 96h 3	45.8 M	41.6 M	26.80%	2.30%	0.19%	90.80%	1.13	50%
HCC827 osimertinib acute 1	45.4 M	41.0 M	24.30%	1.70%	0.20%	90.50%	1.11	51%
HCC827 osimertinib acute 2	54.0 M	48.7 M	25.80%	1.90%	0.19%	90.10%	1.13	51%
HCC827 osimertinib acute 3	54.3 M	49.7 M	25.90%	1.50%	0.19%	91.60%	1.1	51%
HCC827 osimertinib DTP 1	48.5 M	43.2 M	27.00%	2.50%	0.21%	89.10%	1.11	50%
HCC827 osimertinib DTP 2	50.5 M	45.4 M	28.10%	2.60%	0.20%	90.00%	1.15	49%
HCC827 osimertinib DTP 3	55.8 M	51.4 M	27.80%	2.50%	0.19%	92.10%	1.08	50%
HCC827 DTP short washout 1	44.5 M	40.1 M	26.00%	2.20%	0.20%	90.10%	1.09	50%
HCC827 DTP short washout 2	47.5 M	43.4 M	26.50%	2.20%	0.20%	91.40%	1.08	50%
HCC827 DTP short washout 3	63.3 M	58.5 M	28.70%	2.50%	0.19%	92.50%	1.09	51%
PC9 DMSO 1	91.0 M	75.3 M	31.40%	0.60%	0.21%	82.70%	1.11	52%
PC9 DMSO 2	115.3 M	92.1 M	33.10%	0.80%	0.18%	79.90%	1.12	51%
PC9 DMSO 3	95.6 M	79.0 M	32.60%	0.70%	0.19%	82.60%	1.15	51%
PC9 DTP long washout 7d 1	89.1 M	72.3 M	29.60%	0.90%	0.20%	81.10%	1.06	52%
PC9 DTP long washout 7d 2	99.0 M	76.7 M	30.70%	0.90%	0.18%	77.50%	1.06	52%
PC9 DTP long washout 7d 3	91.2 M	74.9 M	31.20%	1.30%	0.18%	82.10%	1.13	51%
PC9 osimertinib acute 1	98.9 M	80.7 M	32.50%	1.70%	0.18%	81.50%	1.12	52%
PC9 osimertinib acute 2	116.5 M	93.2 M	34.10%	2.10%	0.18%	80.00%	1.12	52%
PC9 osimertinib acute 3	99.9 M	82.9 M	32.70%	1.70%	0.19%	83.00%	1.1	52%
PC9 osimertinib DTP 1	88.0 M	70.7 M	29.00%	1.30%	0.20%	80.30%	1.13	52%
PC9 osimertinib DTP 2	103.0 M	85.0 M	32.20%	1.60%	0.19%	82.60%	1.16	52%
PC9 osimertinib DTP 3	112.5 M	92.7 M	31.80%	1.30%	0.18%	82.40%	1.13	52%
PC9 DTP short washout 1	90.4 M	73.1 M	30.60%	1.00%	0.21%	80.80%	1.1	52%
PC9 DTP short washout 2	101.5 M	82.6 M	31.70%	1.10%	0.18%	81.40%	1.16	51%
PC9 DTP short washout 3	102.5 M	85.4 M	33.20%	1.10%	0.19%	83.30%	1.15	52%

Supplementary Table 2: ATAC-seq and ChIP-seq alignment and quality control statistics for DMSO controls and osimertinib DTPs. The percent reads in peaks (RiP) column is a signal to noise metric. We used ENCODE quality control metrics for ATAC-seq including Non-redundant Fraction (NRF) and PCR Bottlenecking coefficients 1 and 2 (PBC1 and PBC2). The interpretation of these metrics are described on ENCODE project website here (https://www.encodeproject.org/atac-seq/).

Assay	Sample Name	Reads	% Reads in Peaks	Avg. Insert Size	PBC1	PBC2	Bottlenecking level	NRF	ENCODE Complexity	Error rate	M Reads Mapped	% Mapped	% Proper Pairs	% GC
ATAC-seq	PC9 DMSO-1	65.6 M	20.78%	107	0.96	28.96	none	0.96	ideal	0.52%	64.7	98.60%	97.70%	47%
ATAC-seq	PC9 DMSO-2	80.6 M	20.14%	108	0.96	27.41	none	0.96	ideal	0.48%	79.5	98.60%	97.70%	48%
ATAC-seq	PC9 DMSO-3	82.1 M	22.49%	106	0.96	27.13	none	0.96	ideal	0.48%	81.1	98.70%	97.90%	48%
ATAC-seq	PC9 osimertinib DTP-1	72.0 M	22.83%	98	0.96	25.29	none	0.96	ideal	0.53%	70.9	98.50%	97.60%	48%
ATAC-seq	PC9 osimertinib DTP-2	80.3 M	23.19%	111	0.96	24.3	none	0.95	ideal	0.51%	79.3	98.80%	97.90%	48%
ATAC-seq	PC9 osimertinib DTP-3	76.1 M	23.24%	108	0.96	24.58	none	0.95	ideal	0.49%	75.1	98.70%	97.80%	48%
ATAC-seq	PC9 osimertinib DTP AZD5153 combo-1	84.2 M	21.07%	100	0.96	25.18	none	0.95	ideal	0.50%	82.9	98.40%	97.40%	48%
ATAC-seq	PC9 osimertinib DTP AZD5153 combo-2	85.5 M	20.01%	100	0.96	23.34	none	0.95	ideal	0.49%	84.1	98.40%	97.50%	48%
ATAC-seq	PC9 osimertinib DTP AZD5153 combo-3	83.9 M	19.37%	96	0.96	23.58	none	0.95	ideal	0.55%	82.7	98.60%	97.60%	48%
ATAC-seq	HCC2935 DMSO-1	67.3 M	27.46%	104	0.97	29.99	none	0.96	ideal	0.37%	66.7	99.10%	98.30%	49%
ATAC-seq	HCC2935 DMSO-2	70.4 M	18.15%	113	0.96	28.45	none	0.96	ideal	0.38%	69.2	98.30%	97.60%	47%
ATAC-seq	HCC2935 DMSO-3	66.9 M	17.90%	114	0.96	28.09	none	0.96	ideal	0.38%	66.1	98.90%	98.20%	47%
ATAC-seq	HCC2935 DTP-1	71.1 M	24.52%	100	0.97	32.01	none	0.97	ideal	0.39%	69.6	97.90%	97.20%	47%
ATAC-seq	HCC2935 DTP-2	83.0 M	28.08%	109	0.96	26.48	none	0.96	ideal	0.36%	82	98.70%	98.00%	47%
ATAC-seq	HCC2935 DTP-3	79.0 M	24.23%	109	0.96	29.36	none	0.96	ideal	0.37%	76.4	96.80%	96.10%	47%
ATAC-seq	HCC2935 osimertinib DTP AZD5153 combo-1	79.7 M	22.69%	101	0.97	30.69	none	0.96	ideal	0.40%	77.8	97.60%	96.90%	48%
ATAC-seq	HCC2935 osimertinib DTP AZD5153 combo-2	84.1 M	30.44%	99	0.95	23.33	none	0.95	ideal	0.37%	83.3	99.00%	98.20%	49%
ATAC-seq	HCC2935 osimertinib DTP AZD5153 combo-3	77.9 M	20.94%	101	0.96	27.98	none	0.96	ideal	0.38%	76	97.60%	96.80%	47%
ATAC-seq	H1975 DMSO-1	83.0 M	35.08%	113	0.94	18.2	none	0.93	ideal	0.38%	82.2	99.00%	98.20%	50%
ATAC-seq	H1975 DMSO-2	90.2 M	37.11%	111	0.94	17.39	none	0.93	ideal	0.36%	89.3	99.00%	98.10%	50%
ATAC-seq	H1975 DMSO-3	88.9 M	35.71%	109	0.94	17.58	none	0.93	ideal	0.40%	87.9	98.90%	98.10%	50%
ATAC-seq	H1975 osimertinib DTP-1	82.8 M	43.53%	149	0.92	13.38	none	0.92	ideal	0.44%	81.6	98.60%	97.70%	50%
ATAC-seq	H1975 osimertinib DTP-2	91.3 M	49.05%	165	0.92	13.82	none	0.92	ideal	0.40%	90.1	98.70%	98.00%	51%
ATAC-seq	H1975 osimertinib DTP-3	88.9 M	51.19%	159	0.92	12.55	none	0.91	ideal	0.41%	87.6	98.50%	97.70%	51%
ATAC-seq	H1975 osimertinib DTP AZD5153 combo-1	80.5 M	48.03%	156	0.92	13.8	none	0.92	ideal	0.40%	79.4	98.70%	97.80%	51%
ATAC-seq	H1975 osimertinib DTP AZD5153 combo-2	87.4 M	47.05%	154	0.91	12.12	none	0.91	ideal	0.40%	86	98.40%	97.60%	51%
ATAC-seq	H1975 osimertinib DTP AZD5153 combo-3	89.8 M	51.12%	150	0.92	13.15	none	0.91	ideal	0.39%	88.7	98.80%	98.00%	52%
ChIP-seq	H1975 pooled input-1	38.2 M	0.00%	0	1	0	severe	1	ideal	0.69%	37.5	97.90%	single-end	42%
ChIP-seq	H3K27Ac H1975 DMSO-1	44.7 M	34.48%	0	1	0	severe	1	ideal	0.68%	43.6	97.60%	single-end	47%
ChIP-seq	H3K27Ac H1975 DMSO-2	42.7 M	45.82%	0	1	0	severe	1	ideal	0.71%	41.9	98.00%	single-end	47%
ChIP-seq	H3K27Ac H1975 DMSO-3	41.1 M	36.36%	0	1	0	severe	1	ideal	0.73%	40.1	97.70%	single-end	47%
ChIP-seq	H3K27Ac H1975 osimertinib DTP-1	41.4 M	35.91%	0	1	0	severe	1	ideal	0.73%	39.8	96.20%	single-end	47%
ChIP-seq	H3K27Ac H1975 osimertinib DTP-2	36.4 M	63.00%	0	1	0	severe	1	ideal	0.89%	35.1	96.40%	single-end	55%
ChIP-seq	H3K27Ac H1975 osimertinib DTP-3	38.6 M	40.86%	0	1	0	severe	1	ideal	0.67%	37.5	97.20%	single-end	46%

Supplementary Table 3: Description of the antibodies, vendor source, item number, and dilution used in Western blot experiments.

Antibody for Western blot	Dilution	Source	Item #
Aurora B	1:1000	Abcam	ab45145
Beta actin	1:10000	Cell Signaling	4970
BIM	1:1000	Cell Signaling	2933
BRD4 [EPR5150(2)]	1:1000	Abcam	ab128874
DUSP4 [EPR19881]	1:500	Abcam	ab216576
E-Cadherin (4A2)	1:1000	Cell Signaling	14472
EGF Receptor	1:1000	Cell Signaling	2232
EGF Receptor	1:1000	Cell Signaling	4267
EGFR [GT133]	1:1000	GeneTex	GTX628887
EpCAM (VU1D9)	1:1000	Cell Signaling	2929
Fibronectin/FN1 (E5H6X)	1:1000	Cell Signaling	26836
GAPDH (D16H11)	1:1000	Cell Signaling	5174
GAPDH (D4C6R)	1:1000	Cell Signaling	97166
IGFBP3 (D1U9C)	1:500	Cell Signaling	25864
MAP2 (D5G1) XP®	1:1000	Cell Signaling	8707
Noggin	1:500	Abcam	ab239520
p38δ MAPK13	1:1000	Cell Signaling	2308
p42/p44 (137F5)	1:1000	Cell Signaling	4695
PAI-1 (SERPINE1)	1:1000	Cell Signaling	11907
Phospho-p42/44 (E10)	1:1000	Cell Signaling	9106
Phospho-EGRF Y1068	1:1000	Cell Signaling	2234
Phospho-Smad1/5/9 Ser463/465	1:500	Cell Signaling	13820
Phospho-Smad2 (Ser245/250/255)	1:500	Cell Signaling	3104
Phospho-Smad3 S423/425	1:500	Cell Signaling	9520
Phospho-YAP S127 (D9W2I)	1:1000	Cell Signaling	13008
SLUG	1:500	Abcam	ab27568
Smad1	1:1000	Cell Signaling	6944
Smad2 (L16D3)	1:1000	Cell Signaling	3103
Smad3	1:1000	Cell Signaling	9523
Smad4	1:1000	Cell Signaling	46535
Smad5	1:1000	GeneTex	GTX60384
TEAD1 (D9X2L)	1:1000	Cell Signaling	12292
Vinculin	1:1000	Sigma-Aldrich	V4139
ZEB1 (E2G6Y) XP®	1:500	Cell Signaling	70512
ZEB2 (E6U7Z)	1:500	Cell Signaling	97885
