# Supplementary material for: The landscape of therapeutic vulnerabilities in EGFR inhibitor osimertinib drug tolerant persister cells
Source: NPJ Precis Oncol. 2022 Dec 27;6:95. doi: 10.1038/s41698-022-00337-w (PMC9794691; doi:10.1038/s41698-022-00337-w)
Supplement: Supplementary file 4 — REPORTING SUMMARY [file 41698_2022_337_MOESM4_ESM.pdf]

nature portfolio

Corresponding author(s): Steven W. Criscione

Last updated by author(s): Sep 19, 2022

Reporting Summary

Nature Portfolio wishes to improve the reproducibility of the work that we publish. This form provides structure for consistency and transparency in reporting. For further information on Nature Portfolio policies, see our Editorial Policies and the Editorial Policy Checklist.

Statistics

For all statistical analyses, confirm that the following items are present in the figure legend, table legend, main text, or Methods section.

n/a	Confirmed	
☐	☒	The exact sample size (*n*) for each experimental group/condition, given as a discrete number and unit of measurement
☐	☒	A statement on whether measurements were taken from distinct samples or whether the same sample was measured repeatedly
☐	☒	The statistical test(s) used AND whether they are one- or two-sided<br>*Only common tests should be described solely by name; describe more complex techniques in the Methods section.*
☐	☒	A description of all covariates tested
☐	☒	A description of any assumptions or corrections, such as tests of normality and adjustment for multiple comparisons
☐	☒	A full description of the statistical parameters including central tendency (e.g. means) or other basic estimates (e.g. regression coefficient) AND variation (e.g. standard deviation) or associated estimates of uncertainty (e.g. confidence intervals)
☐	☒	For null hypothesis testing, the test statistic (e.g. *F*, *t*, *r*) with confidence intervals, effect sizes, degrees of freedom and *P* value noted<br>*Give P values as exact values whenever suitable.*
☒	☐	For Bayesian analysis, information on the choice of priors and Markov chain Monte Carlo settings
☒	☐	For hierarchical and complex designs, identification of the appropriate level for tests and full reporting of outcomes
☐	☒	Estimates of effect sizes (e.g. Cohen's *d*, Pearson's *r*), indicating how they were calculated

Our web collection on statistics for biologists contains articles on many of the points above.

Software and code

Policy information about availability of computer code

Data collection	Custom code was not used in data collection.
Data analysis	No custom code was used in this study. RNA-seq was analyzed using bcbio (v1.2.3), HISAT2 (v2.1.0), Salmon (v0.8.2), and Limma, Voom, ActivePathways, and ClusterProfiler R packages. ChIP-seq and ATAC-seq were analyzed using bcbio (v1.2.3), bwa mem (v0.7.17), samtools (v1.9), biobambam (v2.0.87), deeptools (v3.4), MACS2 (v2.2.6), BEDOPS (v2.4), featureCounts (v2.0.0), ChIPseeker (v1.29.1) and Limma, Voom, ActivePathways, and ClusterProfiler R packages. All statistical analyses were performed in R version 4.1.1.

For manuscripts utilizing custom algorithms or software that are central to the research but not yet described in published literature, software must be made available to editors and reviewers. We strongly encourage code deposition in a community repository (e.g. GitHub). See the Nature Portfolio guidelines for submitting code & software for further information.

Data

Policy information about availability of data

All manuscripts must include a data availability statement. This statement should provide the following information, where applicable:

- Accession codes, unique identifiers, or web links for publicly available datasets
- A description of any restrictions on data availability
- For clinical datasets or third party data, please ensure that the statement adheres to our policy

The RNA-seq, ATAC-seq, and ChIP-seq data used in this study are available through the NCBI GEO database under accession code GSE193259. The remaining data used in this study, including drug screens results, are available in the supporting Supplementary Information Tables.

Human research participants

Policy information about studies involving human research participants and Sex and Gender in Research.

Reporting on sex and gender	N/A
Population characteristics	N/A
Recruitment	N/A
Ethics oversight	N/A

Note that full information on the approval of the study protocol must also be provided in the manuscript.

Field-specific reporting

Please select the one below that is the best fit for your research. If you are not sure, read the appropriate sections before making your selection.

☒ Life sciences ☐ Behavioural & social sciences ☐ Ecological, evolutionary & environmental sciences

For a reference copy of the document with all sections, see nature.com/documents/nr-reporting-summary-flat.pdf

Life sciences study design

All studies must disclose on these points even when the disclosure is negative.

Sample size	The animal studies included a power analysis which included a model variability estimate and an estimate of effect size to be observed. Sample size n=7-9 for most arms was selected to show robust effects on tumor regrowth delay upon drug removal. We were only interested in robust effects on tumor regrowth delays rather than small effect sizes, hence more animals were not suggested by the model.
Data exclusions	There was only one instance of data exclusion. The ATAC-seq data was generated in 4 cell lines including HCC827. However, the HCC827 had lower quality metrics than the other 3 cell lines in the study which was noted in peer review. Due to the lower quality metrics of the HCC827 samples, the Reviewers recommended exclusion of the cell line HCC827 ATAC-seq from the study. We agreed with reviewers assessment and decided to exclude HCC827 ATAC-seq data in the final manuscript.
Replication	The majority of experiments in the study were replicated at least once, including the majority of Western Blots and Immunofluorescence experiments. We have also repeated the RNA-seq and ATAC-seq experiments using slightly different time-points and different batches of the same cell lines. While the drug screen was not repeated, we have individually validated many of the hits in the drug screen using replication dosing studies. The mouse xenograft studies were not done more than once, but were done in multiple models or using multiple drug dosing concentrations to ensure robustness of conclusions. We did not repeat genetic siRNA or CRISPR knockdown studies more than once, however, these themselves were the validation of selective drug inhibitors targeting the same targets. For experiments we have repeated more than once we only report findings that have replicated in multiple experiments. We also comment on negative results observed in some in vivo studies in the manuscript and include studies where findings were negative.
Randomization	For animal studies, mice tumor xenografts were allowed to grow to a specified tumor volume before randomization into treatment or control groups.
Blinding	We did not incorporate experimental blinding procedure in our animal studies. Responses were assessed objectively, and negative study outcomes are also reported in our study.

Reporting for specific materials, systems and methods

We require information from authors about some types of materials, experimental systems and methods used in many studies. Here, indicate whether each material, system or method listed is relevant to your study. If you are not sure if a list item applies to your research, read the appropriate section before selecting a response.

Materials & experimental systems

n/a	Involved in the study
☐	☒ Antibodies
☐	☒ Eukaryotic cell lines
☒	☐ Palaeontology and archaeology
☐	☒ Animals and other organisms
☒	☐ Clinical data
☒	☐ Dual use research of concern

Methods

n/a	Involved in the study
☐	☒ ChIP-seq
☒	☐ Flow cytometry
☒	☐ MRI-based neuroimaging

Antibodies

Antibodies used

See Table S5:
Antibody for Western blot Source Item #
Aurora B Abcam ab45145
BRD4 [EPR5150(2)] Abcam ab128874
DUSP4 [EPR19881] Abcam ab216576
E-Cadherin (4A2) Cell Signaling 14472
EGF Receptor Cell Signaling 2232
EGFR [GT133] GeneTex GTX628887
EpCAM (VU1D9) Cell Signaling 2929
Fibronectin/FN1 (E5H6X) Cell Signaling 26836
GAPDH (D16H11) Cell Signaling 5174
GAPDH (D4C6R) Cell Signaling 97166
IGFBP3 (D1U9C) Cell Signaling 25864
MAP2 (D5G1) XP® Cell Signaling 8707
Noggin Abcam ab239520
p38δ MAPK13 Cell Signaling 2308
p42/p44 (137F5) Cell Signaling 4695
PAI-1 (SERPINE1) Cell Signaling 11907
Phospho-p42/44 (E10) Cell Signaling 9106
Phospho-EGRF Y1068 Cell Signaling 2234
Phospho-Smad1/5/9 Ser463/465 Cell Signaling 13820
Phospho-Smad2 (Ser245/250/255) Cell Signaling 3104
Phospho-Smad3 S423/425 Cell Signaling 9520
Phospho-YAP S127 (D9W2I) Cell Signaling 13008
SLUG Abcam ab27568
Smad1 Cell Signaling 6944
Smad2 (L16D3) Cell Signaling 3103
Smad3 Cell Signaling 9523
Smad4 Cell Signaling 46535
Smad5 GeneTex GTX60384
TEAD1 (D9X2L) Cell Signaling 12292
ZEB1 (E2G6Y) XP® Cell Signaling 70512
ZEB2 (E6U7Z) Cell Signaling 97885

Validation

Antibodies were chosen based on 1) validation in relevant citation in the literature and/or 2) validation experiment from the manufacturer (Cell Signaling or Abcam). We ensured the protein measured were the expected molecular weight by Western. We also conducted multiple knockdown experiments using CRISPR or siRNA or through drug inhibitor target modulation (e.g. osimertinib /phosph-EGFR) that were indirect validation of many of the antibodies used.

Eukaryotic cell lines

Policy information about cell lines and Sex and Gender in Research

Cell line source(s)

PC9, NCI-H1975, HCC827, II-18, HCC4006, HCC2279, and HCC2935 human NSCLC adenocarcinoma cells were obtained from American Type Culture Center (ATCC) and were grown in RPMI 1640, supplemented with 10% FBS, 2 mM L-glutamine, and 1% Penicillin-Streptomycin.

Authentication

Cell lines are maintained in a cell line bank that uses DNA fingerprint profiling for authenticity.

Mycoplasma contamination

Cell lines are maintained in a cell line bank that routinely tests for Mycoplasma contamination.

Commonly misidentified lines
(See ICLAC register)

We did not use any commonly misidentified lines.

Animals and other research organisms

Policy information about studies involving animals; ARRIVE guidelines recommended for reporting animal research, and Sex and Gender in Research

Laboratory animals	Female NCr mice (Charles River Laboratory, US). Female NSG (NOD.Cg-Prkdcscid Il2rgtm1Wjl/SzJ) mice purchased from Jackson Laboratories (Bar Harbor ME) and female SCID or nude mice.
Wild animals	N/A
Reporting on sex	One sex was not considered in the design of the study. Female mice were used in the human tumor cell line or patient derived xenograft studies.
Field-collected samples	N/A
Ethics oversight	Animal studies were conducted in accordance with the AstraZeneca Global Bioethics policy or Institutional Animal Care and Use Committee guidelines and reported following the ARRIVE (Animal Research: Reporting In Vivo experiments) guidelines. For all studies, mice were older than 5 weeks at time of the study.

Note that full information on the approval of the study protocol must also be provided in the manuscript.

ChIP-seq

Data deposition

☒ Confirm that both raw and final processed data have been deposited in a public database such as GEO.

☒ Confirm that you have deposited or provided access to graph files (e.g. BED files) for the called peaks.

Data access links<br>*May remain private before publication.*	The ChIP-seq data used in this study are available through the NCBI GEO database under accession code GSE193259.
Files in database submission	We provide raw FASTQ files, peak Bed files, normalized signal BigWig files, and consensus peak count matrices.
Genome browser session<br>(e.g. UCSC)	We did not load the ChIP-seq to UCSC genome browser.

Methodology

Replicates	Triplicates.
Sequencing depth	~36-44 Million mapped reads single-end.
Antibodies	H3K27ac (Active Motif cat# 39133, lot# 16119013)
Peak calling parameters	NarrowPeaks were called using MACS2 (v2.2.6) using default parameters and IgG sample as a control (Zhang et al., 2008).
Data quality	Chip-seq data quality was addressed using FastQC (https://github.com/s-andrews/FastQC) and multiQC quality tools. Consensus peak counts were filtered by requiring a maximum log2 counter per million >= 2 in any experimental group and peaks called in triplicate in at least one experimental group (removing low depth and non-reproducible peaks). Quality metrics are described in Table S2 of the study.
Software	ChIP-seq and ATAC-seq were analyzed similarly in our study:<br><br>ATAC-seq analysis was performed using the toolkit bcbio (https://github.com/bcbio/bcbio-nextgen) with the hg38 reference Briefly, reads were aligned to hg38 using bwa mem (version 0.7.17). Mitochondrial reads were removed, and alignments were sorted (samtools v1.9), deduplicated (biobambam v2.0.87 bamsormadup), and tn5-shifted (deeptools v3.4 alignmentSieve). ATAC-seq data quality was addressed with FastQC (https://github.com/s-andrews/FastQC), multiQC, and ataqv (Ewels et al., 2016; Orchard et al., 2020). Fragments smaller than 100 base pairs were extracted to generate a nucleosome free (NF) alignment bam file used for consensus peak calling. NarrowPeaks were called using MACS2 (v2.2.6) for both NF aligned reads and separately for all aligned reads (Zhang et al., 2008). Consensus peaks were determined as described in Corces et al. (Corces et al., 2016). Briefly, for all NF NarrowPeaks, a 500bp window was selected around each peak summit. A set of non-overlapping consensus peaks were determined using the maximal scoring peaks as calculated by MACS2 (BEDOPS v2.4, (Neph et al., 2012)). Consensus peak counts were then determined using featureCounts (v2.0.0) counting mapped reads under consensus peak regions (Liao et al., 2014). Consensus peak counts were filtered by requiring a maximum log2 counter per million >= 2 in any experimental group and peaks called in triplicate in at least one experimental group (removing low depth and non-reproducible peaks). Lastly, we annotated peaks with nearest gene using ChIPseeker and focused downstream analysis on peaks within 100 kilobase pairs of a gene transcription start site (Yu et al., 2015). Bigwig signal files of normalized counts per million were also generated using deeptools bamCoverage (v3.4) (Ramírez et al., 2014).<br><br>ChIP-seq analysis was performed as described for ATAC-seq with the following changes, alignments were not Tn5-shifted; and all alignments were used for consensus peak determination.

Differential peak analysis of ChIP-seq and ATAC-seq was performed using the Voom and Limma R package (Law et al., 2014). Cross cell line meta-analysis of H1975, PC9, and HCC2935 differential ATAC-seq peaks was done by intersecting the differential results for individual cell lines using Genomic Ranges (Lawrence et al., 2013). Intersected meta-analysis of ATAC-seq peaks were summarized, for peaks changing consistently across the three cell-lines, by their average fold change and combined adjusted p-value using Fisher's method. We compared ChIP-seq and ATAC-seq to RNA-seq by comparing the fold changes of significant differential peaks, to the fold changes of significantly changing gene expression, for the nearest gene. Pathway analysis on ATAC-seq was done using hypergeometric test using clusterProfiler (Yu et al., 2012). Comparison of differential or consensus peaks to ENCODE SCREEN repository of regulatory elements was done by performing overlaps using Genomic Ranges (Moore et al., 2020).
